# The multiple roles of salt-inducible kinases in regulating physiology

**DOI:** 10.1152/physrev.00023.2022

**Published:** 2023-02-02

**Authors:** Aarti Jagannath, Lewis Taylor, Yining Ru, Zeinab Wakaf, Kayomavua Akpobaro, Sridhar Vasudevan, Russell G. Foster

**Affiliations:** ^1^Sir Jules Thorn Sleep and Circadian Neuroscience Institute, Nuffield Department of Clinical Neurosciences, University of Oxford, Oxford, United Kingdom; ^2^Department of Pharmacology, University of Oxford, Oxford, United Kingdom

**Keywords:** circadian rhythms, immune regulation, salt-inducible kinases, metabolism, sleep

## Abstract

Salt-inducible kinases (SIKs), which comprise a family of three homologous serine-threonine kinases, were first described for their role in sodium sensing but have since been shown to regulate multiple aspects of physiology. These kinases are activated or deactivated in response to extracellular signals that are cell surface receptor mediated and go on to phosphorylate multiple targets including the transcription cofactors CRTC1–3 and the class IIa histone deacetylases (HDACs). Thus, the SIK family conveys signals about the cellular environment to reprogram transcriptional and posttranscriptional processes in response. In this manner, SIKs have been shown to regulate metabolic responses to feeding/fasting, cell division and oncogenesis, inflammation, immune responses, and most recently, sleep and circadian rhythms. Sleep and circadian rhythms are master regulators of physiology and are exquisitely sensitive to regulation by environmental light and physiological signals such as the need for sleep. Salt-inducible kinases have been shown to be central to the molecular regulation of both these processes. Here, we summarize the molecular mechanisms by which SIKs control these different domains of physiology and highlight where there is mechanistic overlap with sleep/circadian rhythm control.

## Graphical abstract

**Figure:**
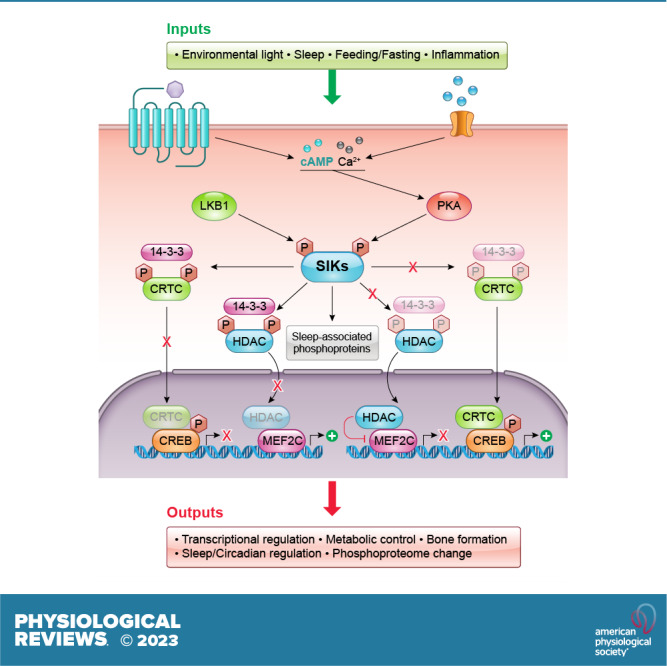

CLINICAL HIGHLIGHTS Salt-inducible kinases (SIKs) have recently emerged as master regulators of multiple aspects of physiology including metabolism, cell division and oncogenesis, inflammation, immune responses, and most recently, sleep and circadian rhythms. In this review, we summarize the molecular mechanisms by which SIKs control these different domains of physiology and highlight where there is mechanistic overlap with sleep/circadian rhythm control. SIK inhibitors have been developed as therapeutics for multiple conditions. These include ovarian cancer, where SIK2 facilitates tumorigenesis by increasing fatty acid synthesis and metastasis; osteoporosis and osteoarthritis, where SIK2/3-mediated transcriptional programs regulate bone resorption; disorders of the immune system, and those of skin pigmentation. In addition, SIK inhibitors hold promise as a potential new therapeutic strategy for neurological disorders such as sleep disorders and depression. Future work to determine the molecular regulation of SIK function and their targets will underpin the development of targeted new therapeutics.

## 1. INTRODUCTION

Salt-inducible kinases (SIKs) are a family of three structurally homologous serine threonine kinases that are part of the AMP-activated protein kinase (AMPK) superfamily. All SIKs are activated by liver kinase B1 (LKB1), which is ubiquitously present and constitutively active, but are deactivated by protein kinase A, which is activated downstream of extracellular signals that elevate cAMP levels by binding to G protein-coupled receptors (GPCRs). SIKs phosphorylate a multitude of targets, the best understood of which are the class 2a histone deacetylases (HDAC4/5/7) and the cAMP response element binding protein (CREB)-regulated transcription coactivators (CRTCs). As such, SIK activity, which is itself dynamic in response to input, modulates transcriptional pathways in response to physiological signals such as dietary sodium, feeding/fasting, and inflammation. Thus the SIK family regulates multiple aspects of physiology, including metabolism, neurophysiology, immune function, and development. Recent work has implicated SIK activity at the hub of a kinase cascade that regulates sleep and circadian rhythmicity, and these processes in turn are master regulators of all physiology and behavior, including metabolism, immune function, and neurophysiology. This review summarizes the state of the art of SIK regulation in multiple areas of physiology, with a focus on how sleep and circadian rhythm regulation, through overlapping pathways centering on the SIK family, could be integrated into these domains.

### 1.1. The Discovery of Salt-Inducible Kinases

The name “salt-inducible kinases” was first coined after the identification of the rapid induction of the said kinase in the rat adrenal gland, following high-salt-diet experimentation in rodents, please see Sun et al. (1) for further details. Cloning identified a peptide of 776 amino acids that bore significant similarity to the protein serine/threonine kinases in the sucrose-nonfermenting 1 (SNF1)/AMP-activated protein kinase (AMPK) family. After the cloning and identification of isoforms through the search of human and mouse genomic databases, this kinase was named *Sik1* (2). Today, this subfamily of proteins consists of three members: SIK1, SIK2, and SIK3 (previously known as SIK, QIK, and QSK, respectively), with SIK2 and SIK3 identified using in silico studies (3, 4). The highest levels of SIK2 can be found in adipose tissues and skeletal muscle, whereas SIK3 is most abundant in the brain (5). All isoforms of SIK are expressed in vertebrates. In invertebrates, an ortholog of mammalian SIK2 is present in *Caenorhabditis elegans* and goes by the name KIN-29. *Drosophila melanogaster* also expresses both SIK2 and SIK3 (6, 7). In humans, SIK1 is located on chromosome 21, while SIK2 and SIK3 genes can both be found on chromosome 11 (8). The proximity of these two genes may allow for their transcription to be coordinated appropriately, further demonstrating how intrinsically linked they are in function. This can also be seen in mice, in which SIK1 is located on chromosome 17, whereas SIK2 and SIK3 are found on chromosome 9. This review summarises the state of the art in our understanding of the regulation and targets of SIKs and their roles in regulating multiple aspects of physiology, as summarised in the table of contents.

### 1.2. Regulation of SIK Expression and Activity

SIK activity changes in response to multiple physiological signals, and this occurs at the level of *Sik* expression and posttranslational modifications. Constitutive expression of *Sik1* appears to be low relative to *Sik2* and *Sik3*, but early studies showed that *Sik1* is rapidly induced by external signals including high dietary salt intake (2) and by adrenocorticotropic hormone signaling (9), membrane depolarisation (10) and glucagon signaling (11). *Sik1* expression is induced by cAMP, whereby the cAMP response element binding protein (CREB) drives the transcription of SIK1 through a highly conserved CRE in the promoter (12). This has been shown in multiple tissue types including in myocytes (13) and the suprachiasmatic nucleus (SCN) in the brain, which regulates circadian rhythms (12). In addition, PKA-mediated phosphorylation of SIK1 allows its nuclear-cytoplasmic shuttling, thus allowing layered temporal control of SIK1 activity by cAMP-PKA signals (14). In contrast, *Sik2* and *Sik3* are constitutively expressed; their mRNA levels do not appear to be induced by cAMP signaling, but their proteins are phosphorylated in response to external signals including cAMP to provide allosteric regulation. The primary kinase that phosphorylates SIK is liver kinase B1 (LKB1), whose phosphorylation of the kinase domain is essential for SIK activation, but the roles for allosteric regulation by CaMK and protein kinase A at other residues on SIK and the evidence for acetylation and ubiquitination are also discussed below.

**FIGURE 1.**
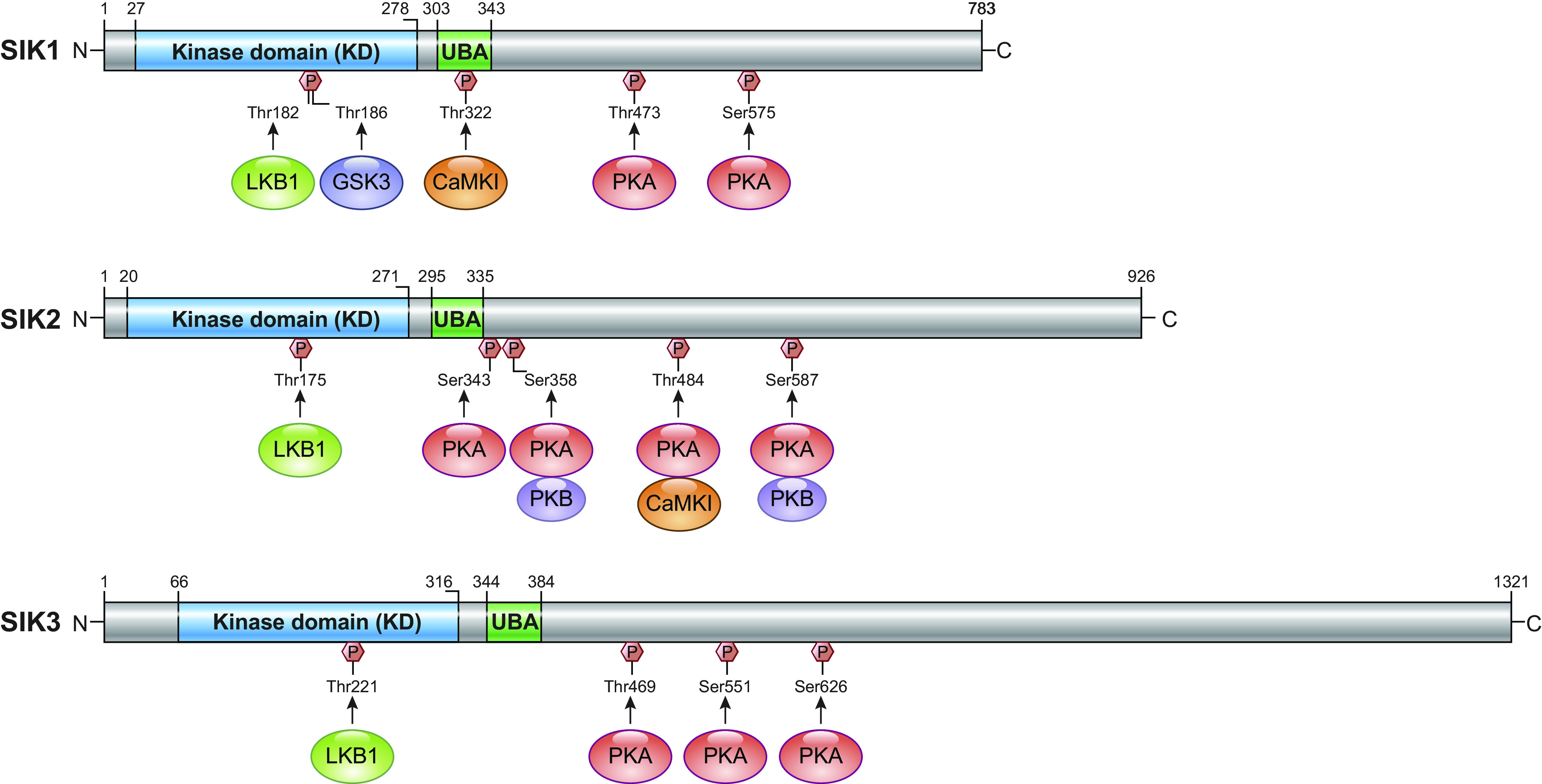
The main structural features of the 3 salt-inducible kinases 1, 2, and 3. The NH_2_-terminal kinase domain with the LKB1 activation site, the UBA domain, and the COOH-terminal domain with multiple PKA regulatory sites are indicated. SIK, salt-inducible kinase; LKB1, liver kinase B1; GSK3, glycogen synthase kinase 3. CaMK1, Ca^2+^/calmodulin-dependent protein kinase I (CaMKI); FOXO1, forkhead box 1; UBA, ubiquitin-associated. Adapted from Ref. 16 per terms of CC-BY Open Access license.

Structurally comparable to AMPK-related kinases, SIKs possess a typical NH_2_-terminal kinase domain, and a ubiquitin-associated (UBA) domain, but also a long COOH-terminal tail that lacks any obvious structural elements and differs between the three isoforms (8, 15) (FIGURE 1). Akin to the family of kinases from which they hail, the phosphorylating activity of all AMPK-related kinases including the SIKs and barring the likes of maternal embryonic leucine zipper kinase (MELK) is dependent upon prior phosphorylation by LKB1 at a threonine residue in the NH_2_-terminal kinase domain’s activation loop (otherwise known as the T-loop) (17). This site is well conserved across the SIKs: located at Thr182 in SIK1, Thr175 in SIK2, and Thr221 in SIK3. Evidence for this key factor in SIK control was provided by studies in LKB1 knockout mice showing that HeLa cells lacking LKB1 activity and expression exhibit significant losses to SIK activity (18–20). LKB1 is considered a master serine/threonine kinase that activates many members of the AMPK-related kinases on the T-loop (18, 21). LKB1 functions as a complex in tandem with the pseudokinase STE20-related adaptor protein (STRADα/β) and the scaffolding mouse protein 25 (MO25), which are localized to the cytoplasm and thus constitutively active and capable of activating SIK (17).

Unlike their AMPK counterparts, the regulation of SIK activity by Ca^2+^/calmodulin-dependent protein kinase (CaMK), of which CaMKK2 is the most versatile, is not observed. While the LKB1 activation site in AMPK is also phosphorylated by CaMK, it was shown that none of the SIKs are activated by CaMKK2 in HeLa cells or extracts (22), and this was replicated in a nonsmall cell lung cancer (NSCLC) model (23). However, the activation of SIK1 downstream of changes in intracellular sodium levels requires phosphorylation by CaMK at Thr322. SIK1 then phosphorylates protein the phosphatase 2A/phosphatase methylesterase-1 (PME-1) complex to ultimately drive the dephosphorylation of the Na^+^-K^+^-ATPase α-subunit, thus increasing its activity and regulating intracellular sodium (24).

**FIGURE 2.**
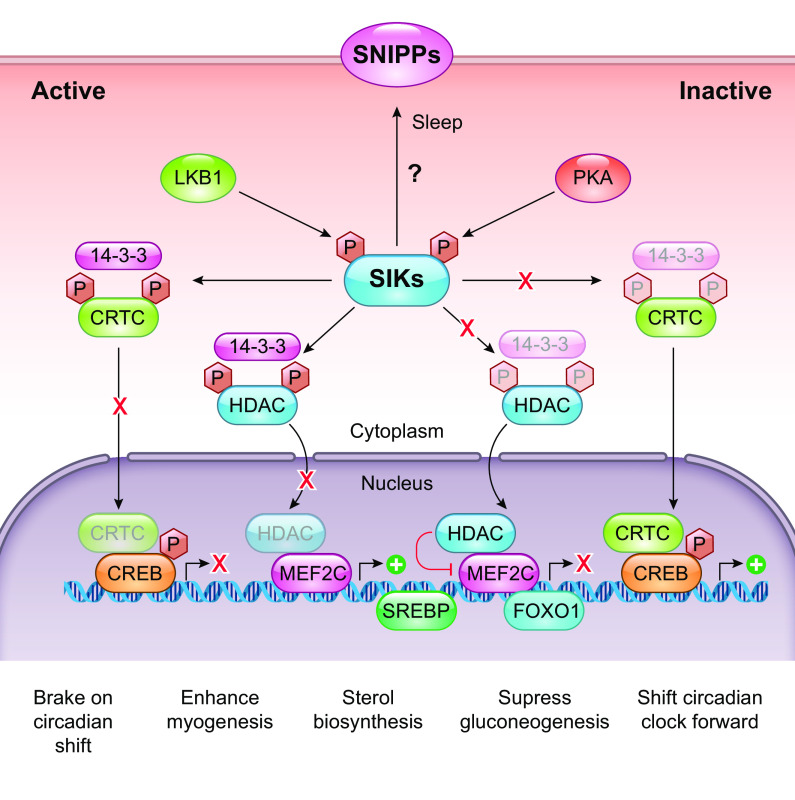
Targets of SIK. When constitutively activated under basal conditions by LKB1, SIKs phosphorylate CRTC and HDAC4/5/7 to result in their sequestration in the cytoplasm by binding to 14-3-3. On phosphorylation by PKA downstream of cAMP signaling, SIKs are inactivated, thus allowing HDAC and CRTC to act on their targets, driving specific transcriptional programs. Precisely how SIKs act on SNIPPs (see sect. 2.1.2) remains unclear. SIK, salt-inducible kinase; LKB1, liver kinase B1; CRTC, cAMP response element binding protein (CREB)-regulated transcription coactivators; HDAC, histone deacetylase; SNIPPs, sleep-need index phosphoproteins; SREBP, sterol regulatory element-binding protein; FOXO1, forkhead box 1. Adapted from Ref. 16 per terms of CC-BY Open Access license.

SIKs have an added layer of phosphoregulation provided by cyclic AMP-dependent protein kinase A (PKA) activity. PKA is a tetrameric enzyme with regulatory (R1/2 α/β) and catalytic subunits (Cα, Cβ, and CG). When cAMP binds to the regulatory subunits, the catalytic subunits are released, which then phosphorylate many downstream targets. Phosphorylation sites for PKA can be found conserved in the COOH-terminal domain: SIK1: T475 S577 (15, 25); SIK2: S343, S358, T484, and S587 (24, 26, 27); and SIK3: T469, S551, and S674) (25, 28). The purpose of this phosphorylation is generally to restrict SIK activity, but the exact mechanisms remain unclear and also differ between the different isoforms. Generally, deactivation of SIK activity is achieved by PKA-enabled binding of SIKs to 14-3-3 proteins in the cytoplasm thus reducing substrate binding. This has been demonstrated experimentally, with a loss of a single 14-3-3 binding site in SIK1 and SIK3 found to be sufficient to render them insensitive to changes in cAMP and eliminate 14-3-3 associations (25). The deletion of PKA promotes SIK1 stability by preventing T475, ultimately slowing its proteosomal degradation (13), but such effects have not been reported for SIK2 or SIK3. Furthermore, under basal conditions, SIK1 is both nuclear and cytoplasmic, but activated SIK1 is shuttled to the nucleus, where it mediates phosphorylation of nuclear targets such as CRTC. However, its phosphorylation by PKA restricts SIK1 to the cytoplasm (14, 29) (FIGURE 2). As such, the application of adrenocorticotropic hormone on Y1 adrenocortical tumor cells results in the nuclear localization on SIK1 within 15 minutes, with cytoplasmic reshuttling occurring over the next 12 hours. When S577 on SIK1 is mutated, SIK1 cannot exit the nucleus, and thus its repression on CREB is increased (14). PKA phosphorylation of SIK2 and SIK3 however does not have the same effect; they remain cytosolic (25, 26). Finally, PKA activates CREB-dependent gene transcription through direct phosphorylation of CREB, as such, doubling down on its anti-LKB1 nature (30).

UBA domains classically facilitate protein degradation by forming motifs to which ubiquitin can bind. However, with respect to SIKs, binding to ubiquitin has yet to be reported. It is suspected that the function of the UBA domain is to assist with LKB1-dependent phosphorylation of the T-loop threonine residue in real time and space, as studies with mutations to UBA domains found decreases in both SIK activation and LKB1 phosphorylation (8, 21). SIK activity is also regulated by acetylation; p300/CBP-mediated Lys-53 acetylation inhibits SIK2 kinase activity, whereas HDAC6-mediated deacetylation restores the activity (31). Finally, autophosphorylation of SIK1 (at Ser186) and possibly SIK2 (at Ser179) has been reported to be essential for sustained kinase activity, granting glycogen synthase kinase 3 (GSK3) access to recognize and phosphorylate the activator Thr182 and Thr175 in a positive feedback loop (32). Phospholipase C (PLC)-inositol (1,4,5)-trisphosphate-dependent Ca^2+^ signaling also results in the autophosphorylation of SIK2 at S358 (33). SIK2 autophosphorylation at S358 regulates its stability in adipocytes (26), but not activity, and this may be of particular relevance in ovarian cancer cells where very high levels of SIK2 are found (33).

### 1.3. Targets of Salt-Inducible Kinases

When active, SIKs influence the phosphorylation state of several targets, through activity at the LxB(S/T)xS*xxxL (B, basic amino acid; X, any amino acid) motif as defined through in vitro studies (34). Of these, two families have been studied extensively: cyclic AMP-response-element binding protein (CREB)-regulated transcriptional coactivators (CRTC1/2/3) and class IIA histone deacetylases (HDAC4/5/7/9). SIK-mediated phosphorylation of class IIa HDACs and CRTC proteins leads to their cytoplasmic retention (35), thus altering downstream transcription (FIGURE 2). Class 2A HDACs remove acetylation marks from histone and nonhistone proteins, thus inhibiting transcription. The phosphorylation and sequestering of class 2A HDACs unburden the transcription factors it would typically be bound to, such as myocyte enhancer factor 2 (MEF2), subsequently promoting MEF2-dependent gene transcription. MEF2 stimulates the expression of muscle-specific genes, thus promoting survival and growth of skeletal and other myocytes and SIK1’s role as a class 2A HDAC in this system has been well studied (13, 23, 29), with the phosphorylation of HDAC4/5/9 freeing MEF2 to transcribe progrowth genes. Under pathological conditions, this ultimately leads to cardiac hypertrophy (36) and calcification (37). However, it was recently shown that the action of SIK1 on HDAC7 leads to its stabilization and increased MYC-mediated transcription (36), suggesting the pathways may be more complex than currently appreciated. In addition, downstream of MEF2 activity lies a wide-ranging influence on the development and differentiation of multiple cell types, including in craniofacial development and bone formation through regulation of DLX5/6 or SOST with subsequent sclerostin expression (38–40) musculoskeletal differentiation through interactions with mastermind-like protein 1 (MAML1) (41) and bHLH transcription factors such as BMAL1 in regulating circadian rhythmicity (42). HDACs also activate forkhead family transcription factors such as forkhead box O (FOXO) and thus regulate glucose homeostasis (43).

Conversely, CRTCs in their dephosphorylated state promote CREB and related basic leucine zipper family (bZip) transcription factor-dependent gene transcription; therefore, cytoplasmic relocalization of CRTC after phosphorylation by SIK acts as a brake on this system. CRTCs are sequestered in the cytoplasm by a phosphorylation-dependent interaction with 14-3-3. The coincidence of calcium and cAMP signaling triggers CRTC dephosphorylation by the phosphatase calcineurin and the inhibition of SIK activity, thus allowing nuclear entry of CRTC (34), since CRTCs promote CREB-dependent gene transcription. SIK is then deactivated by PKA and phosphorylates CRTC to result in cytoplasmic relocalization (26). This pathway has been demonstrated in multiple cell types, with SIK2/CRTC2 in hepatic cells regulating fasting glucose metabolism (11) and pancreatic cells (34) and SIK1/CRTC1 in the SCN (12, 44). The target genes of the transcription factor CREB possess CRE motifs in their promoters and have diverse roles and these are discussed in detail in sect. 2. Briefly, the targets include Period (PER1/2/3) important in the photoentrainment of the circadian cycle (12) and phosphoenol pyruvate carboxykinase (PEPCK) and cytochrome *c*, which assist cellular metabolism and respiration, respectively (45), and several interleukins (2/6/10), TNF-α, and NF-κB, all key players in controlling immunity (46).

The recent development of reliable mass spectrometry-based methods with which to conduct quantitative phosphoproteomics analysis has allowed the identification of at least 100,000 phosphorylation sites on the proteome, if not more (47). However, the kinases mediating these changes and the functional analysis of the phosphosites cover a minuscule fraction of these sites, leading to the term “the dark phosphoproteome” (47). Such approaches have been applied in the study of SIKs, where the phosphoproteome resulting from SIK3 gain of function (S551 mutation that abolishes PKA inactivation) and the effects of the SIK inhibitor HG-9-91-01 were profiled on the whole brain extract (48). This study revealed many changes in phosphorylation resulting from the inhibition of SIKs, but most of these could not be directly attributed to SIK itself, but rather to a cascade of kinases/phosphatases, of which AMPK and several others were suggested to play a major role (48, 49). It is important to note that our current understanding of kinase targets is confined to those that have been well researched; therefore, any annotations of the kinases responsible for phosphosite changes should be made with caution. Nevertheless, a phosphoproteomic analysis of host cell responses to *Salmonella* infection recently picked up an entirely novel target of SIK2: HCT116 cells infected with *Salmonella* surprisingly showed that SIK2 directly associates with actin filaments under basal conditions but, upon *Salmonella* infection, is recruited to the protective *Salmonella*-containing vacuole with elements of the actin polymerization machinery (50).

## 2. THE PHYSIOLOGICAL ROLES OF SALT-INDUCIBLE KINASES

Despite sharing similar structures and targets, the SIK family members play multiple distinct roles in regulating physiological processes centering around the regulation of the best-understood targets HDAC-MEF2C and CRTC-CREB.

### 2.1. The Roles of SIK in Regulating Neurophysiology

All three SIK and CRTC isoforms are expressed throughout the central nervous system and diverse roles for SIK have been described in neuroprotection, depression, epilepsy, sleep, and circadian rhythm regulation, and emerging evidence suggests these roles converge at the level of synaptic physiology regulation.

#### 2.1.1. Circadian rhythms.

**FIGURE 3.**
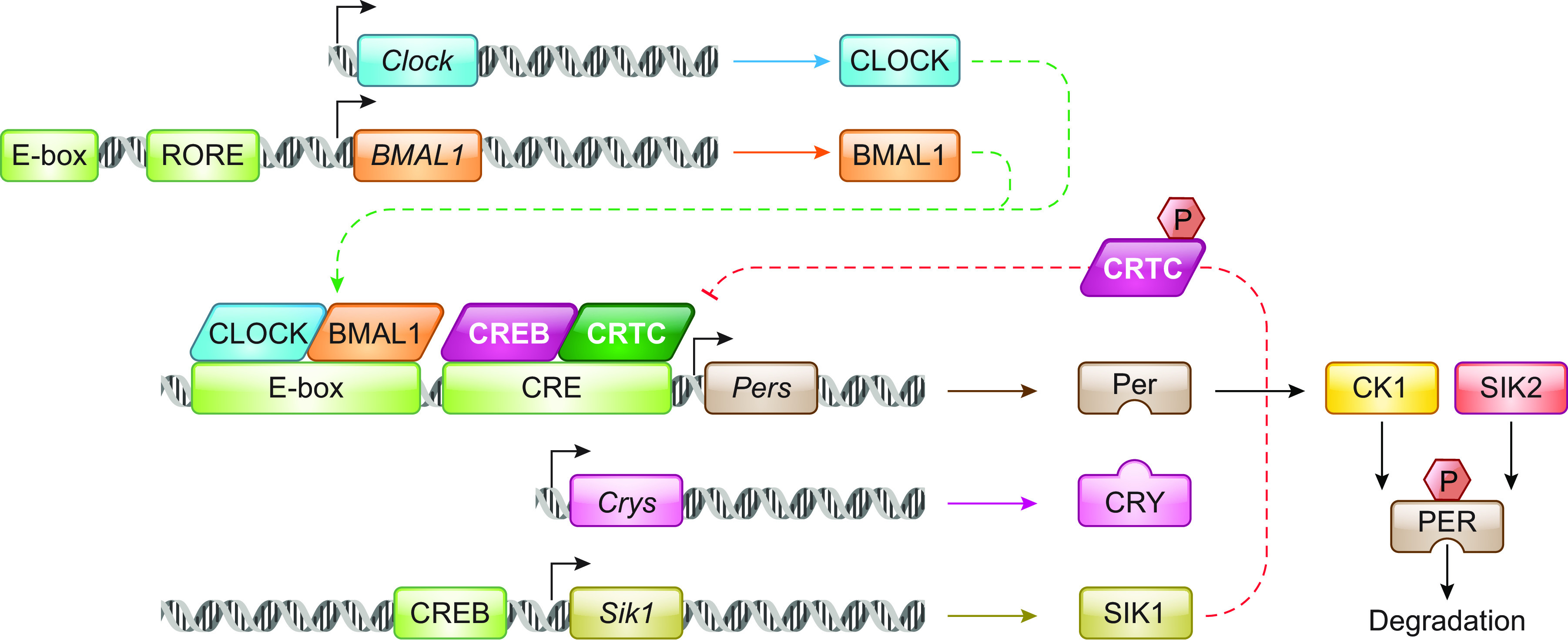
The circadian clock. When constitutively activated under basal conditions by LKB1, SIK phosphorylate CRTC and HDAC4/5/7 to result in their sequestration in the cytoplasm by binding to 14-3-3. On phosphorylation by PKA downstream of cAMP signaling, SIKs are inactivated, thus allowing HDAC and CRTC to act on their targets, driving specific transcriptional programs. Precisely how SIKs act on SNIPPs (see sect. 2.1.2) remains unclear. SIK, salt-inducible kinase; LKB1, liver kinase B1; CRTC, cAMP response element binding protein (CREB)-regulated transcription coactivators; PER, Period; RORE, retinoic acid receptor-related orphan receptor element.

Circadian rhythms are 24-h rhythms in physiology and behavior that are aligned to the external dawn/dusk cycle, and these occur in all domains of life. The circadian clock is found in all cells of the body and is made of a series of interconnected feedback loops that drive rhythmic transcription. At its core, the transcription factors CLOCK and BMAL1 bind to E-boxes located within the promoters of clock-controlled genes, including the repressors PERIOD1/2 (PER) and CRYPTOCHROME1/2 (CRY) (FIGURE 3), which together establishes a 24-h transcription-translation feedback loop (TTFL). The TTFL regulates up to a third of all transcripts in any tissue, thus establishing rhythmicity. Nearly all physiology and behavior show some form of rhythmicity, with the sleep/wake cycle being the most obvious, but blood pressure, metabolism, core body temperature, and hormone secretion are all rhythmic. The TTFL is supplemented by ancillary controls including the positive regulators of BMAL1; the REV-ERBs and the “retinoic acid receptor-related orphan receptors” (RORs) and ubiquitination of CRY by of FBXL family (51). Kinases control circadian rhythmicity at several levels; AMPK directly links energy homeostasis with circadian rhythms by directly phosphorylating CSNK1A and CRY1 (52). The phosphorylation (and subsequent proteosomal degradation) of PER1/2 by casein kinase 1 E and D (*Csnk1e*/d) is one of the primary mechanisms by which the period length of the clock is controlled (53), Indeed, one of the first mammalian clock mutants identified was the *Tau* hamster, which carried a missense mutation in casein kinase I epsilon (*Csnk1e*, *CKIɛ*) and thus exhibited shortened and phase advanced circadian periodicity (54). Furthermore, CRY2 is phosphorylated by the dual-specificity tyrosine-phosphorylation-regulated kinase 1A (DYRK1A) and glycogen synthase kinase 3β (GSK3β), resulting in degradation through the ubiquitin-proteasomal pathway (55).

**FIGURE 4.**
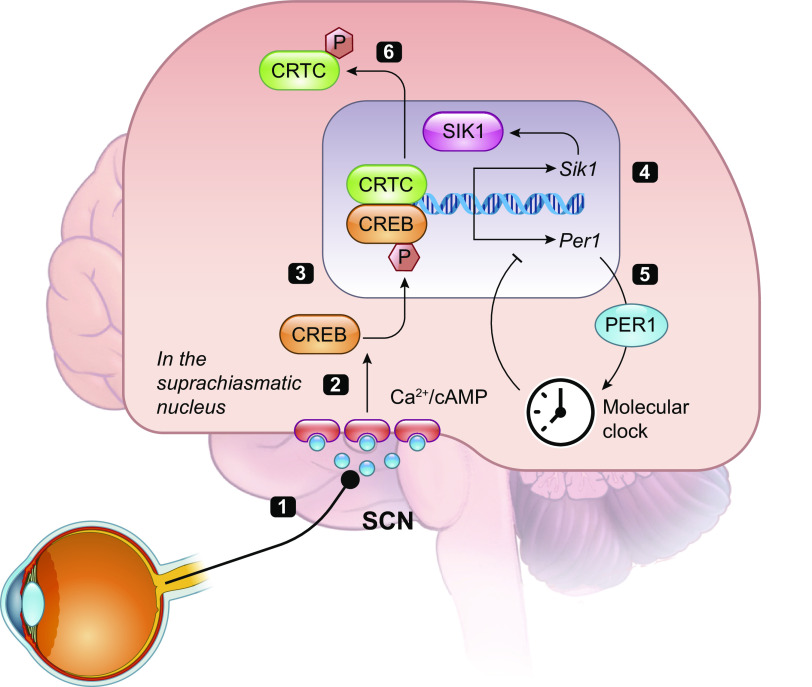
The role of SIK1 in regulating photic entrainment/circadian rhythms. Light input from the retina reaches the SCN (*1*), the master circadian clock, through direct synaptic connections to induce Ca^2+^/cAMP signaling (*2*) within SCN ventral neurons. This results first in the nuclear translocation of CRTC1 and the CREB-mediated transcription (*3*) of clock genes (*5*), which shifts the phase of the circadian clock, as well as Sik1 (*4*). SIK1 phosphorylates CRTC1 to result in its cytoplasmic relocalization (*6*) and a limit on CREB activity. SIK, salt-inducible kinase; SCN, suprachiasmatic nucleus; CRTC, cAMP response element binding protein (CREB)-regulated transcription coactivators; PER1, Period 1.

The circadian clock needs to be aligned to the external light-dark cycle to have any adaptive relevance, and it is in this pathway that a role for SIK1 was first described (12) (FIGURE 4). In mammals, photic input from the retina by the retinohypothalamic tract is conveyed via synaptic connections to the master circadian pacemaker located within the suprachiasmatic nucleus in the hypothalamus, which comprises ∼20,000 densely interconnected neurons (56). Photic input triggers the release of cAMP and Ca^2+^ in response to NMDA and PACAP signals from the retinohypothalamic tract (56), thus resulting in coincidence detection and the dephosphorylation and nuclear translocation of CRTC1 (44). In contrast, photic stimulation does not affect the subcellular localization of CRTC2, which is albeit the less abundant isoform of CRTC in the brain. In addition, PKA-mediated phosphorylation of CREB at Ser133 results in the activation of this transcription factor (57). The resulting CRTC-CREB-mediated transcription drives the expression of clock genes including PER1/2, which alters the phase of the circadian clock, but also of SIK1, which consequently limits CREB transcription through its activity on CRTC1 (12). The participating phosphatase remains to be determined, although multiple phosphatases, including Ca^2+^-activated calcineurin (58) and DUSP4 (59) have been implicated in entrainment. The SCN-specific knockdown of SIK1 removes this inhibitory effect, thus greatly enhancing the response to light in the SCN, resulting in greater phase shifts of the circadian clock and enhanced re-entrainment to a shifted light-dark cycle, which mimics jet lag (12). Importantly, the same transcriptional pathway is also active in cultured fibroblasts in the response to forskolin, indicating SIK1 also regulates the entrainment of peripheral circadian clocks (12). While the precise mechanisms of SIK1 activation and indeed CRTC1 dephosphorylation remain to be elucidated, mice in which the PKA-mediated phosphorylation site of SIK is mutated (Sik1 S577A) showed normal circadian behavior (60), indicating that its deactivation does not have a major regulatory role in this behavior. Subsequently, it was shown that *Sik3*-deficient mice also had defects in circadian rhythms, but these animals showed lengthened circadian rhythms and delayed entrainment to shifted light-dark cycles (61). This was discovered first by studying the metabolic rhythm in these mice, which shows a 6-h delay in the oxygen consumption rhythm (62). It is important to note that *Sik3*-/- mice have multiple birth defects and perinatal death. They exhibit severe hypolipidemia and hypoglycemia (62), defects in chondrocyte hypertrophy, expanded growth plate, and impaired skull bone formation (63). Therefore, phenotypic assessments of this model’s sleep and circadian systems should be made with caution. Nevertheless, individual *Sik3*-/- SCN cells show dampened and more variable rhythmicity as monitored by imaging the PER2-luciferase fusion (64), showing a direct effect of the loss of SIK3 on SCN function. The authors attribute this to the direct phosphorylation of PER2 by SIK3, resulting in its destabilization.

However, a recent study showed that mutations in SIK3 profoundly alter the synaptic phosphorylation landscape of the whole brain (48), and this would undoubtedly impact SCN function and could lead to the deficits in the SCN neuronal function observed in the *Sik3*-/- mice. In contrast to the *Sik3*-/-, mice with a mutation of the PKA-mediated phosphorylation site of SIK3 (S551) showed normal circadian rhythms (60) but uncovered an unexpected role for SIK3 (and also SIK1/2) in the regulation of sleep (65), which was also attributed to the remodeling of the synaptic protein landscape. Given PKA site mutations on Sik3 would result in a gain of function, an assessment of a model harboring the Sik3 loss of function mutation, such as in the LKB1 site, is required to precisely evaluate SIK3’s role in the regulation of circadian rhythmicity.

The SIKs also regulate circadian rhythms in nonmammalian species but not through the pathways described above, suggesting a different evolutionary track. The CRTC1 homolog in *Drosophila* regulates the expression of the gene *Timeless* to maintain 24-h rhythms. The deletion of the *crtc* locus causes period lengthening and amplitude reduction (66). The exact role of the *Timeless* homolog in mammals is unclear, with its equivalent function being performed by *Cry1/2*. A kinase screen for circadian behavior identified that *Sik3* knockdown in a subset of clock neurons known as DN1 neurons in flies causes a reduction in the locomotor period, but a lengthening to the male sex drive period, through its activity on HDAC4 (67). In summary, much remains to be understood about the exact roles of SIK in and their targets regulating circadian rhythms.

#### 2.1.2. Sleep.

**FIGURE 5.**
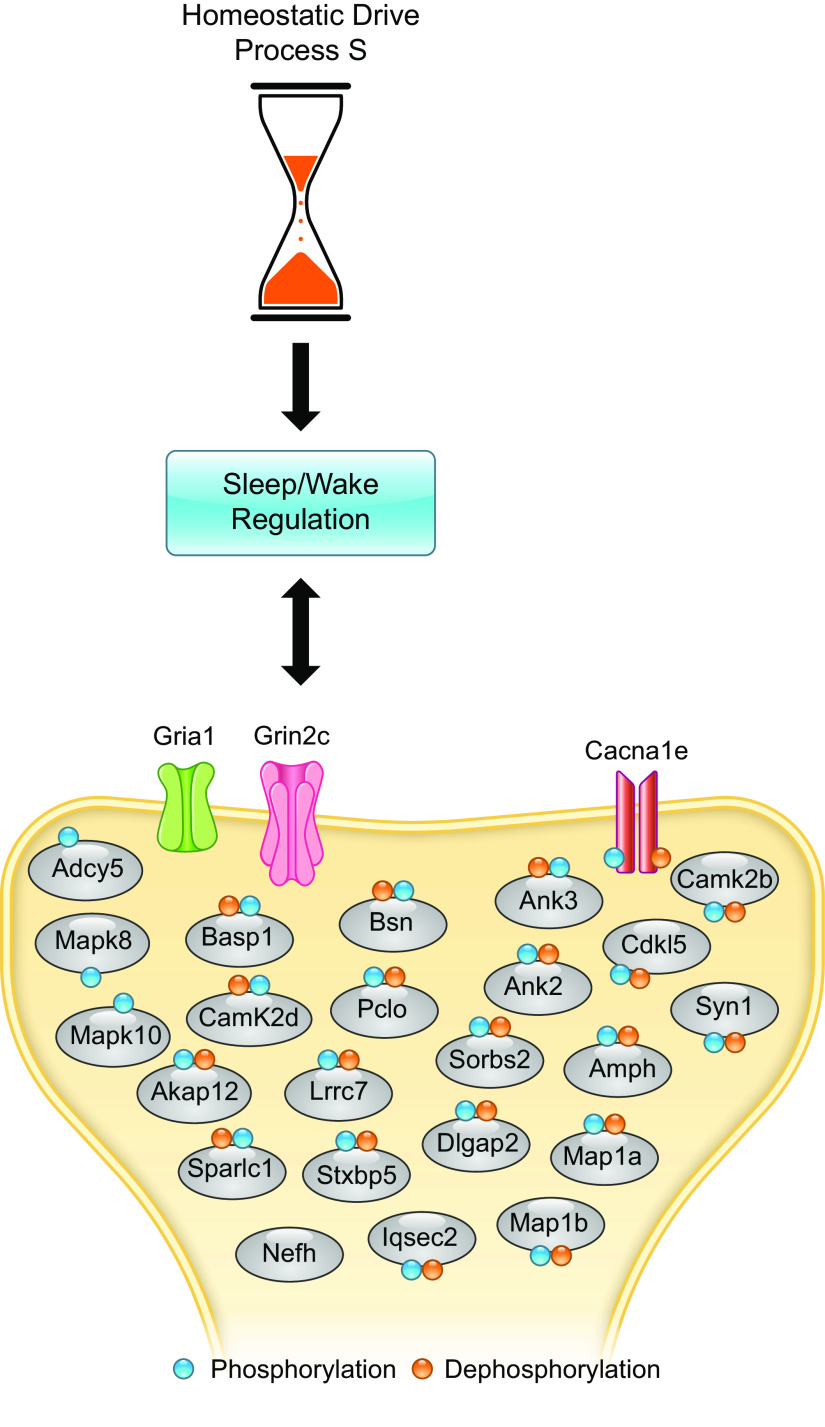
Sleep-regulated protein phosphorylation and synaptic homeostasis. The timing and amounts of sleep are controlled by two main factors, sleep need (sleep pressure/sleep homeostasis), which builds up with time spent awake, and the time of day (circadian rhythms), which gates sleep to the inactive phase. The molecular substrates of sleep need have long remained mysterious, but recent studies place phosphorylation of synaptic proteins by salt-inducible kinase (SIK) at the hub of a network controlling sleep. We hypothesize that the different SIKs are regulated by different input signals (light: signaling to the circadian clock; sleep need: signaling to the homeostat) to provide a unified molecular pathway by which sleep can be modified.

In contrast to circadian rhythms, the mechanisms that underpin the molecular control of sleep are only now emerging. Sleep has long been understood as a state of reduced consciousness, which is different from quiet wakefulness in that the capacity to react to external stimuli is greatly reduced. Despite this being inherently dangerous, sleep is evolutionarily conserved, indicating it serves an incredibly important function. This is thought to be the restoration and repair of the brain after the rigors of wake, through the selective pruning and restoration of synapses, known as the synaptic homeostasis hypothesis (68). However, sleep deprivation has many negative consequences on nearly all aspects of physiology including cognition, metabolism, and immune function (69–71). After more than four decades of research, we still understand very little about how sleep is regulated, and the functions of sleep, at the molecular level. The unknowns in the molecular pathways regulating sleep are best framed in the context of the “Two Process Model.” This provides a behaviorally accurate representation of sleep/wake regulation and states that the sleep/wake transition is regulated by two major drives; the homeostatic drive for sleep (known as Process S), which describes sleep pressure or sleep need and correlates with prior wake, and the circadian drive for wakefulness (Process C) (72, 73). Process C is underpinned by a well-characterized cell-autonomous molecular clock (74) as described above. By contrast, the molecular substrates of Process S remain far more mysterious, as are mechanisms by which Process S and Process C interact to shape the dynamic states of sleep and wake. Many candidates including adenosine have been studied by groups including our own (75), but none have provided a satisfactory mechanistic understanding of sleep homeostasis at the molecular level (76). However, recent studies examining the role of kinases in sleep regulation have begun to transform our understanding of sleep regulation at the molecular level (48, 49, 60, 65, 77) and suggest a plausible molecular readout of Process S in the shape of protein phosphorylation (48, 60, 65, 68, 77–79). One of the key functions of sleep is thought to be “synaptic homeostasis” as discussed above, where net synaptic strength that is potentiated during wakefulness should be downscaled during sleep to maintain plasticity (80–84) (FIGURE 5), but the underlying mechanisms that link synaptic remodeling to sleep are unclear. The findings above suggest synaptic function could be tied to time spent awake through phosphorylation of the synaptic proteins (68).

A large-scale mutagenesis screen was conducted on mice to find genetic determinants of sleep behavior, including sleep need or Process S. This study identified that a mutant in which exon 13 of SIK3 is skipped results in a SIK3 gain-of-function variant, presumably through the loss of the PKA-phosphorylation site contained within this exon. These mice have a greatly increased sleep need, as measured by slow wave activity (SWA) (65); hence, these mice were named *Sleepy*. A similar behavioral phenotype was later observed in site-directed mutants of SIK1–3 (Sik1 S55A, Sik2 S587A, and Sik3 S551A) (60), indicating that when the phosphorylation and thus 14-3-3-mediated sequestration of any of the SIKs is compromised, a sleep phenotype that includes shorter wake time and increased nonrapid eye movement (NREM) sleep time emerges, suggesting a common target profile of all three SIKs that is relevant to the regulation of sleep. Surprisingly, the phosphomimetic mutant SIK3 S551D mouse also shows a similar sleep phenotype (77). The authors suggest this because any changes in the PKA site abolish 14-3-3 binding and this impairs downstream signaling. However, given that the amino acid D should mimic phosphoserine, indicating some discrepancies need to be resolved, an investigation of a true loss-of-function SIK3 mutant would provide a clearer picture. Since much of our knowledge on the function of SIK in sleep regulation comes from studies on mice with the PKA-phosphorylation site of the SIKs being mutated, the use of other models such as those in which the LKB1 regulatory site is compromised (85), or gene knockouts, is required to gain a full understanding of the role of SIKs in the regulation of sleep. As an illustration, these mice with the LKB1 site mutated in SIK1 recapitulate the deficiencies in circadian regulation seen with RNAi-mediated knockdown of *Sik1* (authors’ unpublished data); a PKA-phosphosite mutant does not show any deficits in circadian rhythms (60), indicating the phenotypes resulting from altered activation versus deactivation are markedly different.

The consequences of a mutation in SIK3 were profiled with quantitative phosphoproteomics of the whole brain of the *Sleepy* mouse, or wild-type animals that were sleep deprived or allowed recovery sleep. This study showed that the brain-wide phosphorylation status of key synaptic proteins in mice tracks sleep/wake cycles, with sleep deprivation or greater time awake resulting in overall increased levels of phosphorylation (48, 49) of key synaptic proteins. Wang et al. (48) termed a select subset of differentially phosphorylated proteins (80 in number) as sleep-need index phosphoproteins (SNIPPs). Each of these proteins has multiple phosphosites, numbering in the hundreds for some including the key scaffolding proteins *Bassoon* and *Piccolo*, which are differentially regulated in both directions upon altered sleep state. A subsequent study profiled the phosphoproteome from isolated synaptoneurosomes under different sleep conditions and also saw changes in the phosphoproteome that correlated with sleep need, but surprisingly, a head-to-head comparison of the two databases shows similarity in the proteins phosphorylated, which we will refer to as SNIPPs from here on, but not the sites themselves (author’s unpublished analysis). The SNIPPs show strong enrichment for synaptic scaffolding proteins, neurotransmitter signaling cascade components, and structural elements of axons and dendrites. These include microtubule-associated proteins (MAP1A/B), which are important neuronal structural proteins, ion channels (SCN1A, CACNA1E), NMDA receptor subunits (GRIN2B), kinases (CAMK2B and CDKL5), synaptic vesicle proteins (SYN1), and presynaptic active zone proteins (BSN and PCLO) (48, 49). Taken together, these phosphosite changes are likely to impact synaptic signaling by regulating excitatory glutamatergic signaling and synaptic structural organization, in agreement with the synaptic homeostasis.

**FIGURE 6.**
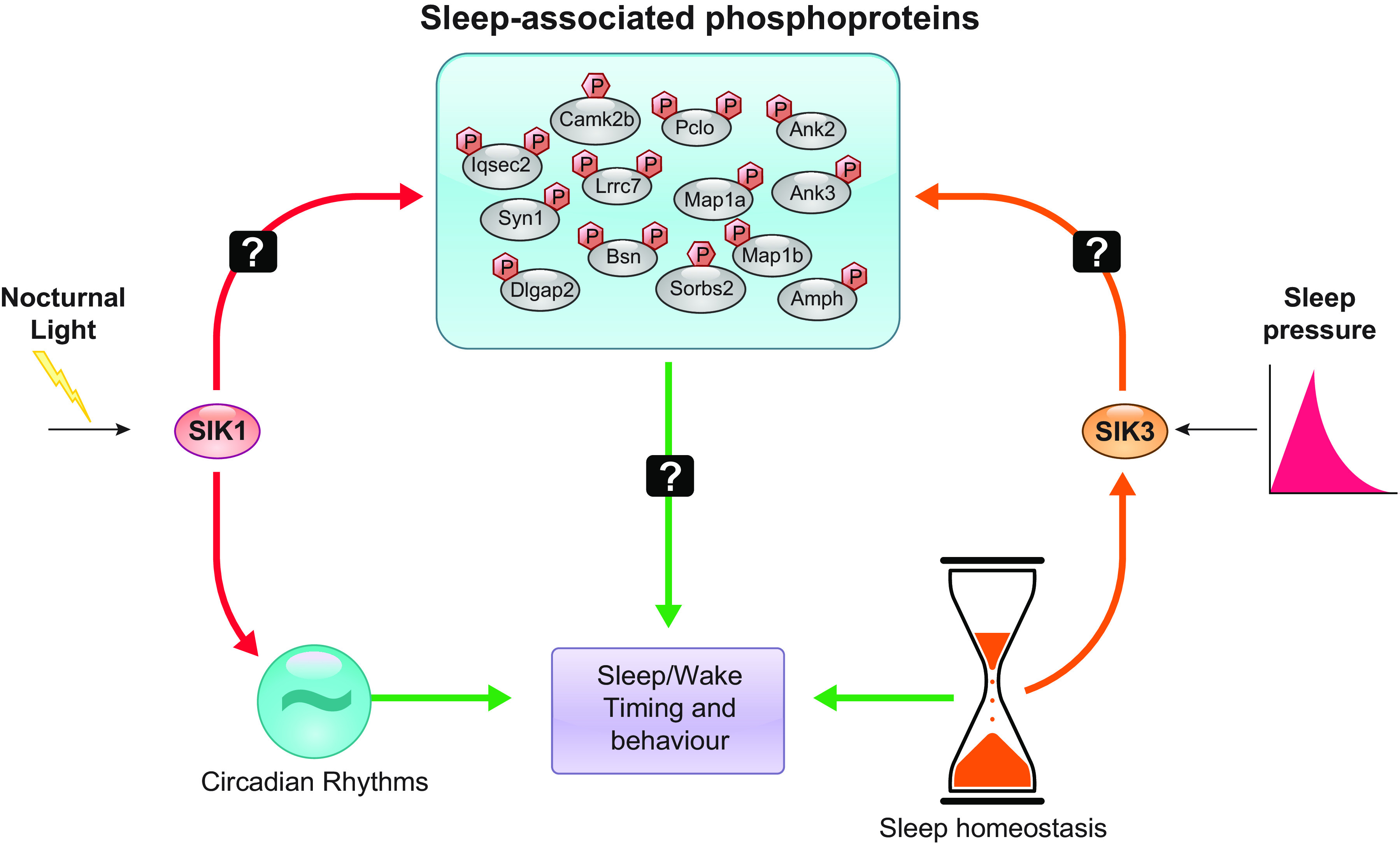
The role of salt-inducible kinase (SIK) in regulating sleep. The timing and amounts of sleep are controlled by two main factors, sleep need (sleep pressure/sleep homeostasis), which builds up with time spent awake, and the time of day (circadian rhythms), which gates sleep to the inactive phase. The molecular substrates of sleep need have long remained mysterious, but recent studies place phosphorylation of synaptic proteins by SIK at the hub of a network controlling sleep. We hypothesize that the different SIKs are regulated by different input signals (light: signaling to the circadian clock; sleep need: signaling to the homeostat) to provide a unified molecular pathway by which sleep can be modified.

There remains much to be addressed in the role of SIK and synaptic phosphorylation in sleep. First, the role of other members of the SIK family remains unclear; studies have shown that preventing the inactivation of any of the SIK isoforms results in a “sleepy phenotype” (60), which reflects the fact that the targets of the different SIK family members are probably similar, but it is the activation of each SIK that is context dependent. Given the role of SIK1 in the photic regulation of the circadian clock (or Process C) is clear, we hypothesize that the SIK family is at the hub of the mechanisms by which both Process S and Process C regulate sleep, but the activation of each SIK is context dependent (FIGURE 6).

Due to the sample requirements in quantitative phosphoproteomics, most studies to date have been in whole brain or large parts of the brain, but sleep is controlled at the circuit level by an array of small brain nuclei with different functions. Precisely where each SIK acts in a spatiotemporal manner is unknown. The phosphoproteomics studies show that the SNIPPs are differentially phosphorylated in response to sleep state at multiple sites and in different amounts/directions. It is unclear which of the SNIPP phosphosites are direct targets of SIK and which result from the activation of other kinases and phosphatases downstream of the SIKs. The activity of a number of kinases has been implicated in sleep control, including CaMKIIα/CaMKIIβ (86) and ERK1/ERK2 (87), with vast representatives of the majority of the major kinase families themselves, including tyrosine kinases, casein kinases and protein kinases A, C, and G, displaying rhythmic phosphorylation that is dependent on the sleep-wake cycle (49), suggesting a kinase cascade operates in sleep regulation, with the SIK family at the center. Furthermore, which of the SNIPP phosphorylation changes are actually required for sleep remains completely unknown. We are of the opinion that rather than a few specific residues, the phosphorylation landscape is what defines the molecular substrate for sleep, such that the thousands of cumulative changes together alter neurophysiology and function in a manner that is conducive to sleep. Quantitative phosphoproteomics is still at the discovery stage, with over 100,000 phosphosites described as collated from curated phosphoproteomics databases, but the kinases for less than 5% have been identified and the function for far fewer is known (47).

While the current state-of-the-art has demonstrated a link between salt-inducible kinases, synaptic protein phosphorylation, and sleep regulation, future work will address the distinct and redundant contributions of each SIK to sleep and circadian regulation, their spatiotemporal sites of action, and the consequences of SIK’s action on the SNIPPs in terms of synaptic and brain physiology. The phosphorylation of SNIPPs will doubtless have effects on neuronal excitability and neurotransmission, yet this has not been explored yet in *Sik* transgenic mouse models to date. However, it was shown recently in *Drosophila* that SIK3-regulated gene expression controls the glial capacity to buffer K^+^ and water, which is an important mechanism by which glia regulate neuronal excitability (88). Two very recent studies suggest that the phosphorylation of HDAC4 and possibly HDAC5 by SIK3 regulates the levels of NREM sleep (89, 90), as heterozygous *Hdac4^S245A^* mice, in which the SIK3 target is mutated, show reduced NREM time, and overexpression of HDAC4(S245A) using the adeno-associated virus AAV-PHP.eB in *Sik3* (*Sleepy*) mice rescued the hypersomnia phenotype (89). Furthermore, upregulated transcription of synaptic genes in glutamatergic cortical cells was driven by the gain of function of SIK3 or sleep deprivation, which correlated with an increase in EEG delta power during NREM sleep (89). Similar results were reported by Zhou et al. (90), where they used a chimeric knockout of LKB1 kinase, which is required to activate SIK3. However, neither of these studies related their findings to the synaptic phosphoproteome, leaving how HDAC4/5-regulated transcriptional control ties in with synaptic phosphorylation, and thus synaptic function, an open question. Importantly, these and the other studies have clearly demonstrated that SIKs provide the framework by which multiple external cues can be transduced to regulate gene expression and protein phosphorylation, which ultimately regulate sleep. Sleep and circadian rhythms are master regulators of physiology, with their disruption affecting all physiological processes including metabolism, cell cycle control/oncogenesis, and immune function. Given SIKs also regulate these processes independently of the clock, there are multiple overlapping regulatory mechanisms at play centering around kinase control. These mechanisms are considered throughout this review.

#### 2.1.3. Epilepsy.

Exome sequencing has identified mutations in the SIK1 gene in developmental epilepsies, which is consistent with the important roles for SIK1 targets including MEF2C and CRTC1/CREB transcriptional target genes in neurodevelopment and epilepsy pathogenesis (91–93). This is also consistent with the recently identified roles of the SIK family in shaping the synaptic phosphoproteome (see sects. 2.1.1. and 2.1.2), which could profoundly impact neurophysiology. Furthermore, BDNF increases SIK1 expression in primary rat cortical neurons, which results in enhanced MEF2 transcription of multiple genes regulating neurophysiology including Arc and Nur77 (94). One hundred one individuals with early myoclonic encephalopathy, Ohtahara syndrome, and infantile spasms, but no identified causative mutations were sequenced, and of these, six were unrelated individuals had mutations in SIK1, of whom three had missense mutations and three COOH-terminal truncations in the nuclear localization domain. The mutations were all outside the main kinase and regulatory domains; indeed, SIK1 was capable of autophosphorylation and HDAC5 phosphorylation in all cases, but in vitro assays demonstrated that the mutations resulted in increased protein stability and/or altered cellular localization (95). These individuals presented with neonatal epilepsy and short survival or with autism following infantile spasms. Two following studies described the changes in neurophysiology resulting from such truncations. Badawi et al. (96) generated a SIK1 mutant mouse with a COOH-terminal truncation with CRISPR/Cas9-mediated genome editing and recapitulated the altered nuclear/cytoplasmic localization seen with the human mutations. These mice showed an increase in excitatory synaptic transmission and increased excitability in layer 5 cortical pyramidal neurons and also an increase in repetitive behavior and social behavioral deficits. Proschel et al. (97) overexpressed either wild-type or 4 different mutant SIK1 isoforms [as described in Hansen et al. (95)] in human primary fetal cortical neurons and showed that epilepsy-causing mutations in SIK1 were associated with decreased MEF2C protein and transcriptional activity, and decreases in the length and number of neurites. Together, these findings support the ideas presented in sect. 2.1.2, where the role of SIK in regulating synaptic neurophysiology has been discussed. Interestingly, one of the patients described in Hansen et al. showed disrupted sleep. More recent work confirms the further identification of SIK1 mutations and resulting alterations MEF2C regulation in pediatric epilepsies (98).

#### 2.1.4. Neurotrophin signaling.

The roles of SIK in regulating neurophysiology other than those described above center around the regulation of CREB and its downstream targets. Given the ubiquitous roles of Ca^2+^ and cAMP in nearly all neuronal signaling, the phosphorylation of CREB and resulting changes in gene expression have been described as underlying the long-term changes in dendritic morphology (99) synaptic architecture and plasticity, and neurodevelopment (100–102). CRTC1 has been described as a cofactor for CREB in several of these processes. Membrane depolarization induces CRTC1 dephosphorylation and nuclear accumulation, followed by the induction of SIK activity and CRTC phosphorylation, in the SCN as described above (44) but also cortical neurons (92), hippocampus (103), hypothalamus (104), and amygdala (105). This activity is region and context specific; for example, contextual fear conditioning causes the nuclear translocation of CRTC1 in the basolateral amygdala alone but not the hippocampus (105). Stress induced by restraint results in the induction of *Sik1* mRNA in corticotropin-releasing hormone (CRH) neurons in the hypothalamus, which in turn deactivates CRTC allowing rapid control of CRH transcription (106). Interestingly, stress only activates SIK1, but not SIK2, as seen with circadian rhythms. However, SIK2 appears to control CRTC nuclear/cytoplasmic shuttling in CRH neurons under baseline conditions (104) and also downstream of NDMA signaling in cortical neurons (107), thus regulating protection from excitotoxicity. SIK2 degradation is crucial to the activation of CRTC1 and the resulting neuroprotection after ischemia (107). Much of the neuroprotective effect of CRTC-CREB has been attributed to the induction of brain-derived neurotrophic factor (BDNF), along with other immediate early genes including *Fos*, *Arc*, and *Egr1*. BDNF is a widely expressed neurotrophic factor that promotes neuronal survival, differentiation, and synaptic plasticity and growth (108). Every 5′-exon of the BDNF gene is regulated by its own promoter, with CREB playing a crucial role in its transcriptional control. BDNF positively regulates its own expression by signaling through the TrkB receptor; this function was shown to not require CRTC activity (109); however, the activity-dependent transcription of BDNF has been shown to involve CRTC1 (105, 110). CRTC1, through the regulation of BDNF, has been implicated in multiple neurodegenerative and neuropsychiatric conditions (111, 112), but the evidence for major depressive disorder is presented below. A number of studies have shown that the deficiency of either CREB or BDNF in the hippocampus results in depression-like behaviors in rodents (113–116). *Crtc1* knockout mice display depression-like phenotypes in behavioral tests including the forced swim test, increased social withdrawal and decreased sexual motivation (117), and resistance to the effects of commonly used antidepressants such as fluoxetine and desipramine, which fail to increase *Bdnf* transcription as observed in wild-type mice. Chronic social defeat-induced stress (CSDS) and chronic mild stress (CMS) are both used to model depression in mice and induced SIK2 in the hippocampus and prefrontal cortex and, in agreement, also reduced nuclear CRTC1. Overexpression of SIK2 in the hippocampus induced depression-like behaviors, accompanied by attenuated BDNF signaling and neurogenesis, while knockdown of SIK2 produced antidepressant-like effects (103). The same group followed up with the administration of the brain-permeant SIK inhibitor ARN-3236 to show that this compound reversed the effects of CSDS and CMS in behavioral readouts and BDNF signaling/neurogenesis (118). Given the effects of SIK described above on synaptic protein phosphorylation, and the close mechanistic links that connect sleep, circadian rhythms, and depression (119), the exact targets of SIK that mediate antidepressant action remain to be determined. Nevertheless, this pathway represents a significant advance over the traditional monoamine-targeting pharmacological routes. While these compounds have remained the mainstay of treatment in neuropsychiatric conditions, their efficacy is limited and there is a pressing need for novel target development.

### 2.2. Adaptation to Salt Intake/Sodium Sensing

As mentioned previously, SIK1, the founding member of this subfamily, was identified as induced in the adrenal gland of rats fed a high-salt (HS) diet and thus named salt-inducible kinase 1 (2). It was subsequently shown to be central to the adaptation to variations in intracellular sodium in a number of cell types, by an indirect action that ultimately results in the dephosphorylation of Na^+^-K^+^-ATPase, increasing its activity (24). Na^+^-K^+^-ATPase is an active transporter known for its role in pumping sodium and maintaining resting membrane potential and cell volume. Its role has been described in many cell types including intestinal and renal cells, neurons, and diverse aspects of physiology including the regulation of blood pressure. Therefore, the loss of SIK1 should lead to decreased Na^+^-K^+^-ATPase activity, and accordingly, *Sik1* knockout (KO) mice on a high-salt diet developed higher systolic blood pressure and cardiac hypertrophy and also showed hypertensive phenotypes (120). This was shown to be associated with deregulated transforming growth factor-β1 signaling (121) and E-cadherin expression, thus destabilizing intercellular junctions (122). No differences in serum renin and angiotensin II levels were observed, but they did display an overdrive of sympathetic nervous system signaling (123), thus suggesting the pathways may be more complex than suggested. In contrast, SIK2 KO mice do not show increases in BP in response to a high-salt diet or changes in plasma Na^+^ and K^+^ levels but display cardiac hypertrophy in response to high-salt intake (124). Double knockouts have led to further confounding results; SIK1/2 double knockout mice surprisingly show an increase in blood pressure but no hypertrophy (125). Furthermore, the modulation of Na^+^-K^+^-ATPase activity in renal proximal tubules in the kidney by SIK (primarily SIK1) is responsible for sodium reabsorption; thus SIK1-KO mice but not SIK2 KO display increased natriuresis (121, 124). In summary, SIK is an important regulator of the electrolyte homeostasis, with the different members playing unique roles.

### 2.3. Metabolism

SIK kinase activity is influenced by biological changes, such as energy depletion and insulin or glucagon perturbation (5), placing SIKs at the center of a network that responds to hormones and regulates metabolic pathways that maintain glucose and lipid homeostasis (FIGURE 3). The SIKs are subordinate to the AMPK family, known for their role in sensing changes in energy and regulating metabolism on a cellular level, balancing ATP production with consumption (126). The identification of LKB1 as an upstream regulator of AMPK, and another 12 AMPK-related kinases including the SIK family, demonstrated a complex regulatory network relying on multiple kinases influenced by external signals including hormones and behavior.

#### 2.3.1. Glucose uptake.

Glucose is taken up by cells as the main source of ATP production. This process is predominantly dependent on the expression and/or recruitment of sodium-dependent glucose transporters (SGLT) and glucose transporters (GLUT). GLUT4, expressed in adipose tissue and skeletal muscles, mediates the majority of glucose uptake in response to insulin (127). Importantly, SIK2 seems to play a significant role in positively regulating glucose uptake in adipose tissue as GLUT4 expression in adipocytes has been shown to be suppressed by SIK2 substrates (128–130). CREB in concert with CRTC2/3 has been shown to downregulate GLUT4 expression and promote insulin resistance. This occurs by CREB-mediated upregulation of the transcriptional repressor activating transcription factor 3 (ATF3) (128, 131). Furthermore, overexpression of SIK2 increases glucose uptake while pharmacological inhibition reduces it (130). In human adipose tissue from obese individuals, SIK2 and SIK3 mRNA were downregulated. Of interest, SIK2 mRNA levels negatively correlated with in vivo insulin resistance, while its protein and kinase activity negatively correlated with body mass index (132). Together, this supports a role for SIK2 in protecting against obesity-induced insulin resistance, particularly at the adipose-tissue level. On the other hand, SIK1 seems to exert an opposing effect on insulin responses in skeletal muscles, inhibiting glucose uptake and promoting insulin resistance. This is likely a result of direct phosphorylation of the insulin receptor substrate 1 (IRS1) (133, 134). Indeed, SIK1 KO mice on a high-fat diet do not exhibit hyperglycemia or increased gluconeogenesis but display pronounced improvement in peripheral insulin sensitivity, glucose tolerance, and skeletal muscle glucose uptake as a result of upregulation of GLUT1, GLUT4, and GLUT12 expression (133).

In pancreatic beta cells, similarly opposing roles for SIK1 and SIK2 on insulin secretion have been reported. On one hand, SIK1 has been shown to reduce insulin release by phosphorylating and activating phosphodiesterase 4D, the enzyme responsible for cAMP breakdown, leading to a reduction in cellular cAMP levels and an attenuation of insulin secretion (135). By contrast, SIK2 has been shown to promote insulin release. SIK2 was reported to phosphorylate the cyclin-dependent protein kinase 5 (CDK5) activator p35, which presents it for ubiquitination and proteasomal degradation. Consequently, the decline in p35 levels represses the activity of CDK5, which in turn reduces its inhibitory phosphorylation of the voltage-dependent Ca^2+^ channels (VDCC). This promotes an increase in Ca^2+^ influx that potentiates exocytosis and the release of insulin from its storage vesicles (136). Together, this emphasizes the distinct functions and tissue-specific actions of SIK1 and SIK2 in regulating glucose uptake.

#### 2.3.2. Gluconeogenesis.

Gluconeogenesis is a metabolic pathway that generates glucose from noncarbohydrate substrates (such as amino acids, lactic acid, etc.). This process is tightly regulated by insulin and glucagon, which alter the expression levels of the first and last catalytic enzymes in the pathway, glucose-6-phosphatase (G6P) and phosphoenol-pyruvate carboxykinase (PEPCK), respectively (5, 137). Proliferator-activated receptor-γ coactivator-1α (PGC-1α) also plays a role, upregulating G6P and PEPCK gene expression and enhancing hepatic gluconeogenesis as a result (138). Of note, PGC-1α is a nodal target of the cAMP/PKA/CREB axis (139) and can be suppressed by insulin via AKT-mediated forkhead box 1 (FOXO1) phosphorylation (140).

SIK family members have been implicated in gluconeogenesis (16). In primary hepatocytes, loss of SIK1 increased mRNA levels of the gluconeogenic enzymes PEPCK, PGC-1α, and G6P, while overexpression of SIK1 suppressed gluconeogenesis, by increasing CRTC2 phosphorylation and consequently inhibiting cAMP-induced PEPCK expression (11). Furthermore, SIK1 knockdown in mice induced gluconeogenic gene expression and fasting hyperglycemia (11). Together, these observations propose an inhibitory role of SIK1 on gluconeogenesis. Similar findings were reported with SIK2 and SIK3 (141–143). SIK2 has been identified as a downstream target of the PI3K/AKT pathway that mediates insulin responses, whose role as a suppressor of gluconeogenesis is well established (141). Indeed, insulin treatment induces SIK2-mediated phosphorylation and ubiquitin-dependent degradation of CRTC2 (141). Like SIK1, SIK3 knockout in hepatocytes has also been linked with the upregulation of the gluconeogenic genes PGC-1α, PEPCK, and G6P (143).

The mechanisms underlying these SIK-mediated effects on gluconeogenesis are unclear in some respects and remain under investigation. One key pathway is via CRTC2 phosphorylation, which restricts it to the cytoplasm and promotes its degradation (16, 25). This in turn inhibits CRTC’s gluconeogenic role as a binding partner of CREB and a transcriptional inducer of gluconeogenic gene expression like PEPCK, PGC-1α, and G6P (5, 144), thus allowing SIK to suppress gluconeogenesis. Another is by the PKA-mediated phosphorylation and inactivation of SIK2 (142) and possibly also SIK3 (143) in response to glucagon, as the glucagon receptor is a GPCR that is coupled to PKA-CREB signaling, thus allowing glucagon to prevent suppression of gluconeogenesis by SIK. On the other hand, other studies have suggested the involvement of class 2a HDACs, whereby the activity of SIK blocks their nuclear translocation (5, 16) and suppresses the deacetylation/activation of FOXO-mediated PEPCK and G6P expression, thereby inhibiting gluconeogenesis via HDAC phosphorylation (43).

Despite the strong involvement of SIKs in regulating gluconeogenesis via different pathways, some studies have reported discrepant findings on the role of SIK1 and SIK2 in vivo. Data from a liver-specific SIK1 knockout demonstrated that SIK1 is not needed for CRTC2 phosphorylation and gluconeogenesis regulation in vivo (133). Another study demonstrated that SIK2, but not SIK1, plays a vital role in feeding and insulin-mediated inhibition of gluconeogenesis (141). However, this was followed by two independent reports that examined SIK2-deficient hepatocytes in vivo and demonstrated a lack of effect on gluconeogenic gene expression and glucose levels (131, 142). However, interestingly, broad pharmacological inhibition of SIK isoforms (using the pan-SIK inhibitor HG-9-91-01) was shown to dephosphorylate CRTC2/3 and HDAC4 and significantly enhance gluconeogenic gene expression and glucose formation in hepatocytes (142). This effect was abolished when LKB1 was genetically eliminated or an inhibitor-insensitive SIK mutant was introduced, suggesting a clear role for the LKB1-SIK pathway as a gluconeogenic suppressor in the liver and proposing a dispensable role for SIK2 in this pathway. On the other hand, SIK3 seems to be a key player. Under lactate-induced gluconeogenesis in a fed state (where insulin suppresses gluconeogenic pathways), SIK3, but not SIK1 or SIK2, KO mice exhibit a rapid increase in blood glucose levels following lactate administration, suggesting that loss of SIK3 isoform induces constitutive activation of gluconeogenesis (143).

Collectively, it is evident that SIK activity is necessary for the inhibition of gluconeogenesis and that the LKB1-SIK pathway functions as a gluconeogenic gatekeeper in mouse liver. However, the contributions of various SIK isoforms in vivo and the precise mechanisms underlying these functions are yet to be elucidated.

#### 2.3.3. Lipid metabolism.

Lipids are essential sources of energy that are tightly regulated by complex networks governing lipogenesis, lipolysis, and fatty acid oxidation. They also form the building blocks for steroid hormones such as estrogen, progesterone, and adrenocorticotropic hormones (145). Increasing evidence proposes SIK1 and SIK2 as negative regulators of lipogenesis, albeit via different mechanisms. Overexpression of SIK1 suppresses hepatic expression of key lipogenic genes, such as acetyl-CoA carboxylase (ACC) and fatty acid synthase (FAS), rate-limiting steps in fatty acid synthesis, whereas knockdown of SIK1 significantly enhances their expression (146). This inhibition of lipogenic gene expression is likely a result of direct phosphorylation and inhibition of the sterol regulatory element-binding protein (SREBP-1c) at the Ser329 site by SIK1, given coinfection of mutant SREBP-1c with SIK1 transgenic restored hepatic triacylglycerol levels and lipogenic gene expression caused by SIK1 overexpression (146). However, this study did not show phosphorylation of the endogenous SREBP-1c at SIK sites; therefore, a deeper investigation of SREBP-1c as a bona fide substrate of SIK1 is still lacking. Furthermore, the use of alternative models (such as SIK1 knockout) is of importance and could verify the mechanistic role of SIK1-mediated SREBP-1c phosphorylation in hepatic lipogenesis.

Similar to SIK1, SIK2 also inhibits hepatic lipogenesis (147), with *Sik2* highly expressed in brown adipose tissue (BAT) (3, 142). The carbohydrate-responsive element-binding protein (ChREBP) is a transcriptional activator of glycolytic and lipogenic genes, whose activity is upregulated by the histone acetylate transferase (HAT) coactivator p300. SIK2 was shown to phosphorylate p300 on Ser89, thereby inhibiting its activity and suppressing ChREBP-mediated hepatic lipogenesis (147). In adipocytes, SIK2 has also been shown to attenuate insulin-mediated lipogenesis via phosphorylation of IRS-1, while SIK2 depletion in preadipocytes enhanced their adipogenic potential in a CRTC2-dependent manner (7, 131). The different mouse models used provide conflicting results about the exact role of SIK2; *Sik2*-deficient mice showed body weights similar to those of wild-type mice (124). However, mice that are mutated for the PKA-phosphorylation site of SIK2 (S587A) residue specifically in BAT were susceptible to diet-induced obesity (148). Possible explanations include a redundant function of other SIKs in the former model but SIK2 overactivity in the second. Together, the inhibitory role of SIKs on lipogenesis is evident and seems to occur via different pathways in a tissue-specific manner, although further studies are required to resolve apparent discrepancies.

#### 2.3.4. Steroidogenesis.

SIKs also play a role in steroidogenesis (4, 7). Steroidogenic acute regulator protein (StAR) and cytochrome P450 cholesterol side chain cleavage (P450scc) are two core enzymes in steroid synthesis (145, 149). StAR controls the transport of cholesterol across the mitochondria membranes, while P450scc enzyme (coded by the CYP11A gene) catalyzes the formation of the steroid precursor pregnenolone from cholesterol (149). In cultured adrenocortical cells, adrenocorticotropin (ACTH) treatment induces a rapid but transient expression of SIK1, which precedes an upregulation of P450scc and StAR steroidogenic enzymes. However, overexpression of SIK1 in these cells results in a significantly lower basal level of CYP11a mRNA, which fails to respond to ACTH induction. Similarly, StAR mRNA, although still upregulated in the presence of ACTH, had a significantly lower induction relative to control cells (9). This suggests that SIK1 might act as a repressor, not an activator, of steroidogenic gene transcription in these cells. Subsequent studies demonstrated that SIK1 inhibits CREB transcriptional activity, thereby repressing the CRE-mediated transcription of StAR and CYP11A gene in adrenocortical cells (7, 149, 150). Together, this suggests that SIK1 plays a role in fine tuning the initial phase of steroidogenesis by regulating the expression of steroidogenic enzymes.

SIK isoforms have also been shown to affect different aspects of lipid homeostasis, as SIK2 has been shown to promote cholesterol synthesis (151). SIK2 upregulates SREBP2 expression and, in turn, enhances transcription of the cholesterol-synthesizing enzyme 3-hydroxy-3-methyl-glutaryl-coenzyme A reductase (HMGCR) (151). Loss of function studies have also shed light on the role of SIK2 in regulating fatty acid oxidation in the liver and skeletal muscles. Park et al. (131) found that SIK2 KO mice demonstrated a significant reduction in key enzymes responsible for fatty acid oxidation, such as carnitine palmitoyl-transferase 1 (CPT-1), peroxisomal acyl-CoA oxidase (ACOX1), and mitochondrial medium chain acyl COA dehydrogenase (MCAD). On the other hand, SIK3 was shown to promote lipid storage in *Drosophila* by inhibiting HDAC and CRTC activity (16, 152). In mice, it was also shown to alter energy storage by regulating cholesterol, bile acid metabolism, and fatty acid synthesis, whereas SIK3 KO mice demonstrated significant inhibition of the latter (62). Nonetheless, evidence from mammals supporting the role of SIK3 in regulating lipid metabolism is not well validated.

Taking all observations into consideration, the SIKs clearly play a vital role in regulating lipid metabolism; however, the mechanisms underlying SIK-mediated actions on lipid metabolism are still not well understood. Thus more elaborative investigations of the distinct roles of the SIK family members in regulating lipid metabolic homeostasis are needed.

### 2.4. Circadian Regulation of Metabolism

**FIGURE 7.**
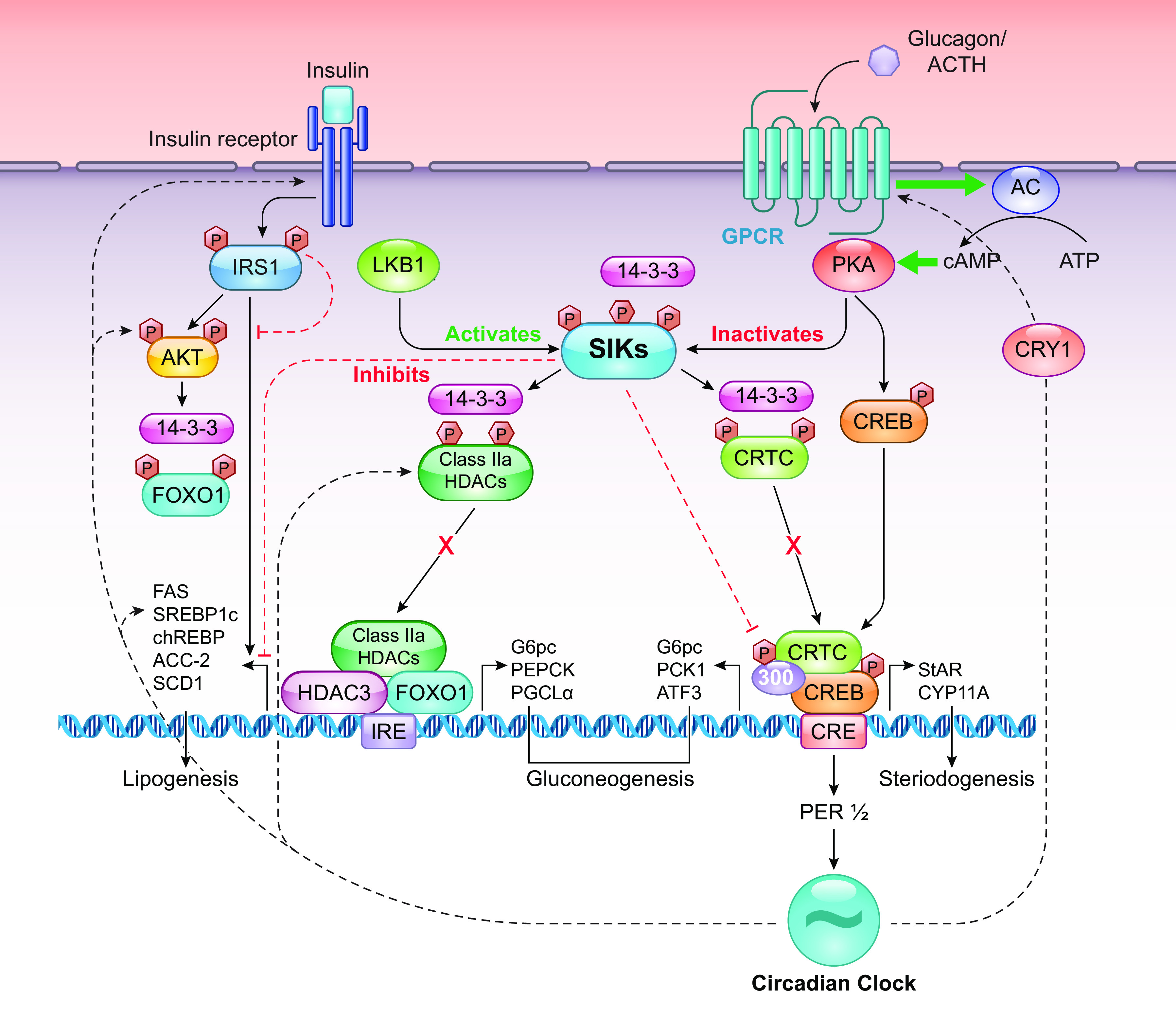
The effects of SIKs on glucose metabolism. SIKs play a crucial role in regulating glucose and lipid metabolism. Through their regulation of CRTC and HDAC activity, SIKs repress gene expression and inhibit gluconeogenesis, lipogenesis, and steroidogenesis. SIK can also phosphorylate IRS1, thereby inhibiting insulin-mediated actions. Furthermore, SIK2 suppresses the transcription repressor ATF3, which consequently enhances GLUT4 expression and promotes glucose uptake. SIK1 also represses the clock gene PER1, and this shift in the clock can feedback to the regulation of signaling at the glucagon receptor, insulin receptor, insulin secretion, and Akt phosphorylation. SIK, salt-inducible kinase; GPCR, G protein-coupled receptor; FOXO1, CRTC, cAMP response element binding protein (CREB)-regulated transcription coactivators; HDAC, histone deacetylase; IRS1, insulin receptor substrate 1; FOXO1, forkhead box 1; GLUT4, glucose transporter 4; PER1, Period 1; ATF3, activating transcription factor 3; PGC-1α, proliferator-activated receptor-γ coactivator-1α; PEPCK, phosphoenol pyruvate carboxykinase; ACC-2, acetyl-CoA carboxylase; FAS, fatty acid synthesis; LKB1, liver kinase B1; StAR, steroidogenic acute regulator protein; SREBP, sterol regulatory element-binding protein. Adapted from Ref. 1 per terms of CC-BY Open Access license.

Nearly every element of metabolic regulation is under circadian control, whereby the SCN and feeding rhythms both provide time cues to the peripheral organs, resulting in rhythmic transcription. An analysis of the liver transcriptome shows that over 30% of the transcripts cycle has a 24-h rhythm, and these are implicated in multiple metabolic pathways (153, 154) such as glucose, cholesterol, and triglyceride metabolism. In addition to the core TTFL elements, clock-controlled genes in turn exert their effect to bring about rhythmic transcription. These include the D site of albumin promoter (albumin D-box) binding protein (DBP), which regulates insulin and hepatic leukemia factor (HLF) and thyrotroph embryonic factor (TEF), which regulates thyroid-stimulating hormone release. The ancillary loop of the clock, which includes the Rev-erb (REV-ERB) receptor family, retinoic acid orphan receptors (RORs), peroxisome proliferator-activated receptors (PPARs), and other nuclear receptors (NRs), in turn, have all been shown individually to regulate genes associated with glycogen, fatty acid, and triglyceride metabolism. These target genes include glycogen synthase, HMG-CoA reductase which is the rate-controlling enzyme that produces cholesterol; CYP7A1 is a rate-limiting enzyme in bile acid synthesis, acetyl-CoA carboxylase (ACC), and FAS. As a result, clock gene mutant mice display multiple metabolic abnormalities. For example, homozygous *Clock* mutant mice (*Clock*^Δ19/Δ19^), which show a loss of function of this core clock gene, are obese and hyperphagic and develop hyperglycemia, hyperinsulinemia, hepatic steatosis, and dyslipidemia (155). Metabolic pathways in turn feedback on the circadian clockwork. Restricting feeding to daytime (sleep phase) in mice causes uncoupling of peripheral clocks within the liver, kidney, heart, and pancreas from SCN rhythms (156, 157), with feeding providing the dominant time cue in place of light to peripheral clocks. The molecular pathways underlying the transmission of these time cues remain to be fully elucidated; however, it is possible the SIK family plays a role, mirroring its role in transducing light information to the SCN. SIK activity would directly modify the expression of *Per1/Per2*, which in turn would modify the phase of expression of other clock genes. This shift in rhythmicity would then manifest where the clock directly regulates metabolism. For example, CRY1 directly modulates the activity of the glucagon receptor (158), and the expression of the insulin receptor, the insulin secretory pathway (159), and the phosphorylation of AKT are all rhythmic (160). Thus SIKs, which sense feeding/fasting stimuli, could directly feedback to the clock to regulate the phase and strength of oscillation of the clock, thus providing the framework for immensely complex regulatory networks, with multiple interlocking feedback loops (FIGURE 7).

### 2.5. The Role of SIK in Cancer

New therapeutic targets and pathways remain a key focus in oncology research, and there have been several exciting advances around the SIK family. They have been found to play a significant role in tumorigenesis, and the three members of the SIK family show distinct expression and regulatory patterns.

LKB1, which is one of the upstream regulators of SIK and AMPK, is a commonly mutated gene in lung and cervical carcinomas, functioning as a tumor suppressor in part due to its action on AMPK, which itself is a tumor suppressor (161). In tumor progression, metastasis is categorized as a late stage when the oncogenic growth spreads to a secondary site of the body. For metastasis to occur, cancer cells must resist anoikis, which is a form of apoptosis following detachment from the extracellular matrix (ECM), that is mediated by tumor protein 53 (p53). Cheng et al. (161) discovered that SIK1, activated by LKB1, is an essential upstream regulator of p53-mediated anoikis, and thus suppresses metastasis (162). Using a CRISPR/Cas9-mediated approach, this tumor-suppressing activity of the LKB1-SIK1 axis was also demonstrated in a mouse model of oncogenic KRAS-driven lung adenocarcinoma (163). Furthermore, reduced expression of SIK1 is associated with the faster appearance of distal metastasis, that is cancer spreading to distal organs or lymph nodes, and poor prognosis in human breast cancer (161, 162, 164). Furthermore, SIK1 modulates metastasis by regulating the epithelial-mesenchymal transition (EMT) process, which is an essential pathway for tumor cells to gain mobility and metastasize (165). In addition to increased metastasis, the loss of SIK activity also reprograms transcriptional pathways in a pro-oncogenic manner; Ródon et al. (166) showed that a loss of LKB1 inactivates SIKs, which leads to the cytoplasmic shuttling of CRTCs and increased CREB-mediated transcription. This significantly contributes to tumor cell proliferation and chemotherapy medication resistance (166). Finally, as major regulators of metabolic pathways, SIKs present attractive targets in countering the metabolic reprogramming that happens in cancer. SIKs regulate aerobic glycolysis, which is a process that supplies energy for tumor cell proliferation. Ponnusamy et al. (167) reported that SIK1, downregulated in breast cancer, suppresses aerobic glycolysis, while SIK3 showed opposite effects. In agreement, other studies have confirmed significantly reduced expression of SIK1 in breast cancer, which inhibits p53 activity as SIK1 is an upstream regulator (161, 165). In addition to acting as a mediator in the metastasis pathway, p53 facilitates oxidative phosphorylation and suppresses aerobic glycolysis by negatively regulating Glut1 and lactate dehydrogenase A (LDHA). On the other hand, increasing glucose levels activate SIK3/mammalian target of rapamycin (mTOR) activity, which augments cancer cell proliferation and aerobic glycolysis (167, 168). Interestingly, the SIK3/mTOR signaling pathway rescues cancer cells from cell death by suppressing the SIK1/p53 pathway, suggesting complex interplay even within the SIK family. Indeed, in triple-negative breast cancer tissues, SIK3 is highly expressed while SIK1 and SIK2 are reduced (165). Double knockouts of SIK1 and SIK3 increase triple-negative breast cancer invasion, which agrees with the finding that SIK1 and SIK3 are involved in LKB1-mediated tumor suppression pathways (18, 23, 163, 166). Mice with the triple knockout of SIK1, SIK2, and SIK3 displayed increased resistance to chemotherapy including paclitaxel and cisplatin (165). Together, these findings suggest a novel marker in SIKs for cancer prognosis and prediction for chemotherapy resistance.

**FIGURE 8.**
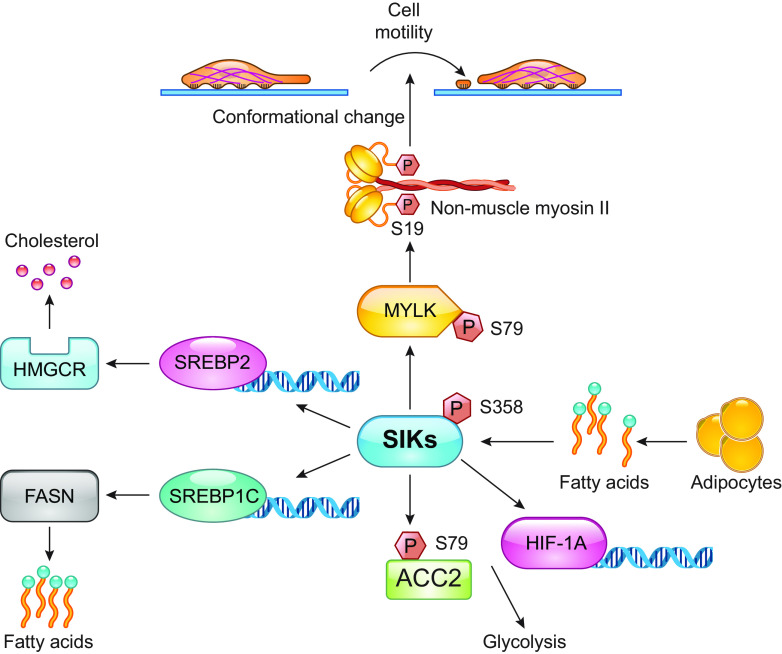
The role of SIKs in ovarian cancer. SIKs play a crucial role in regulating glucose and lipid metabolism. Through their regulation of CRTC and HDAC activity, SIKs repress gene expression and inhibit gluconeogenesis, lipogenesis, and steroidogenesis. SIK can also phosphorylate IRS1, thereby inhibiting insulin-mediated actions. Furthermore, SIK2 suppresses the transcription repressor ATF3, which consequently enhances GLUT4 expression and promotes glucose uptake. SIK1 also represses the clock gene PER1, and this shift in the clock can feedback to the regulation of signaling at the glucagon receptor, insulin receptor, insulin secretion, and Akt phosphorylation. SIK, salt-inducible kinase; CRTC, cAMP response element binding protein (CREB)-regulated transcription coactivators; HDAC, histone deacetylase; IRS1, insulin receptor substrate 1; GLUT4, glucose transporter 4; PER1, Period 1; ATF3, activating transcription factor 3; HMGCR, 3-hydroxy-3-methyl-glutaryl-coenzyme A reductase; LKB1, liver kinase B1; HIF-1A, hypoxia-inducible factor-1A; FASN, fatty acid synthase; SREBP, sterol regulatory element-binding protein; ACC-2, acetyl-CoA carboxylase;

SIK2 is emerging as an interesting target in ovarian cancer, and the underlying pathways have been investigated as follows. Chen et al. (169) reported that the downregulation of SIK1 leads to higher levels of microRNA 141 (miR-141), which is a noncoding RNA that plays an important role in gene expression and chemotherapy sensitivity in ovarian cancer cells, and that increased expression of SIK1 inhibits cancer cell proliferation. Compared with SIK1, which is expressed highly in the adrenocortical gland, adipose, and neural tissues, SIK2 is expressed mainly in the adipose in human organs. Ovarian cancer is highly metastatic and often presents in the omentum, an adipocyte-rich tissue in the abdominal cavity, and the overexpression of SIK2 is observed in ovarian cancer metastasis (33, 131, 151, 170). Adipocytes encourage fatty acid oxidation and ovarian cancer cell proliferation by producing free fatty acids in the omentum (171) and ovarian cancer cells (151). The overexpression of SIK2 increases cholesterol and fatty acid synthesis by upregulating the sterol regulatory element binding proteins (SREBP) transcription factors SREBP1c/FASN and SREBP2/HMGCR (151). SREBP1c in turn regulates fatty acid synthase (FAS) and SREBP2 3-hydroxy-3-methylglutaryl-CoA reductase (HMGCR), both enzymes involved in tumor cell growth (151). Furthermore, SIK2 also regulates and is in turn regulated by, glucose metabolism (142), an oncogenic increase. SIK2 upregulation is activated by the phosphoinositide 3-kinase (PI3K)-Akt signaling pathway, which regulates glucose metabolism, and the overexpression of this pathway leads to cancer cell proliferation (172, 173). Gao et al. (174, 175) suggested that SIK2 then in turn drives glycolysis by upregulating the expression of transcription factor hypoxia-inducible factor-1α (HIF-1α), which modulates the adaptation of cancer cells to hypoxic environments. Adipocytes activate SIK2 via the phospholipase/Ca^2+^ transduction pathway, which augments PI3K-Akt signaling and acetyl-CoA carboxylases (ACC) phosphorylation in ovarian cancer cells (33). As a component in fatty acid synthesis, upregulation of isoform ACCα is observed in multiple cancers and is therefore considered a new potential target for cancer therapy (176). In addition, metastasis is a process that involves increased cell motility such as actin polymerization and adhesion on the cell surface. By interacting with filamentous actin (F actin), nonmuscle myosin II (NMII) plays an important part in cell movement, which is regulated by the phosphorylation of myosin light chain 2 (MYL2) at Ser19. SIK2 overexpression in human ovarian cancer cells increases F-actin expression and MYL2 phosphorylation, which significantly promotes ovarian cancer metastasis (177, 178). These data propose that SIK2 contributes to tumor cell proliferation by participating in multiple overlapping processes that together facilitate tumorigenesis (FIGURE 8). In support, SIK2 knockout increases the sensitivity of ovarian cancer cells to paclitaxel and SIK2 expression is higher in paclitaxel-resistant cancer cells (179).

As a result, there is extensive literature on the discovery of novel and specific inhibitors of SIK2 as a primary treatment or secondary treatment to enhance chemotherapeutic efficacy for ovarian cancer. Arrien Pharmaceuticals developed novel 1H-(pyrazol-4-yl)-1H-pyrrolo[2,3b] pyridine inhibitors, ARN-3236 and ARN-3261, that suppress SIK2 activity by competing with ATP binding. In ovarian cancer cells, ARN-3236 inhibits centrosome splitting and AKT activation, which are pathways involved in metastasis (180). It also significantly enhances the sensitivity of ovarian cancer cells to paclitaxel (180) as does ARN-3261 to platinum-based drugs such as carboplatin (181). Raab et al. (170) have recently developed a G-5555 derivative small molecule inhibitor, MRIA9, that is potent and SIK2 selective, to interrupt the adipocyte-mediated tumor cell progression pathway. SIK2 activity is associated with the autophosphorylation level of S358, a key residue regulating SIK2 protein stability (33), and MRIA9 significantly reduced SIK2 S358 autophosphorylation, SIK2 activity, and ovarian cancer cell metastasis. Through siRNA screening, SIK2 was also identified as a regulator of the G_2_/M phase in the cell cycle and found to be localized in the centrosome (170, 179). As a result, inhibition of SIK2 causes spindle assembly and centrosome function failure, which leads to improper spindle orientation and genomic instability for ovarian cancer cells (179). Microtubule-independent mitosis inhibitors have started to emerge as a therapeutic approach, but the effects and toxicity remain unclear. Extending to clinical applications, Taylor et al. (182) showed that CA125, a tumor-associated antigen (TAA), could differentiate between the early and late stages of ovarian cancer. Despite CA125 being a high-potential biomarker, serum CA125 is not observed in most patients with advanced ovarian cancer (183). In addition, Charoenfupraset et al. (184) reported SIK3 as a novel TAA as it is involved in the regulation of LKB1’s effects in that increasing expression of SIK3 in OVCAR3 human cancer cells promoted G_1_/S cell cycle transition and cell growth. Finally, in recent years, cancer immunotherapy (CIT), a treatment that increases T-cell (TC) activation and cytotoxicity (185), has been trialed in ovarian cancer. However, the high occurrence of resistance to CIT greatly interferes with therapeutic efficacy. Sorrentino et al. (186) discovered that SIK3, overexpressed in tumor tissues, counteracts TC cytotoxicity by upregulating TNF-induced nuclear factor-κB (NF-κB). This suggests that SIK3 is a possible endogenous mediator of resistance against TC-regulated cytotoxic molecules. Therefore, inhibition of SIK3 can potentially reduce tumor growth and invasiveness (186). In light of these findings, it is suggested that combinations of biomarkers with SIK3 would aid prognosis and that SIK3 could also provide the substrate for new therapeutic applications in ovarian cancer.

Research has shown that disruption of the circadian clock is intimately linked to cancer, metabolism, and cell cycle control, to the extent that shiftwork is classified as a Class2a carcinogen (187), and this has been reviewed extensively (188–192). Furthermore, strengthening the clock, or working with the clock, can also slow cancer progression (191, 193). The SIK family could well lie at the interface of the pathways linking the two; all three SIKs regulate oncogenesis, metabolism, and sleep and circadian rhythms. Direct evidence for the role of SIK in mediating circadian control in cancer is lacking, but several lines of evidence point to deregulated clock gene expression in cancer. One point of convergence is on the PER genes, where the SIK family regulates both their transcription and protein stability (see sect. 2.1.1). Gery et al. (194) reported that PER1 is associated with ATM serine/threonine kinase and Chk2, which function in DNA damage response. PER genes are deregulated at many levels in cancer, such as altered methylation of PER1 and PER2 promoter (195, 196) and increased expression, for example, in prostate cancer, as PER1 is regulated by androgen receptor signaling (197). In agreement, evidence from Kubo et al. (198) and Conlon et al. (199) supported that shift work and sleep disruptions significantly increase risks for prostate cancer in chosen Japanese and Canadian cohorts, respectively. In breast tumors, Winter et al. (200) discovered that the decrease in expression of PER1 in familial breast cancer is particularly significant, and the low expression of PER1 is related to negative estrogen receptor (ER) levels, which make hormone therapy drugs ineffective. Interestingly, Yang et al. (201) described breast cancer growth as a cycle that is regulated by PER1, with two daily peaks and troughs. The downregulation of PER1 increases the circadian amplitude of the daily peaks, which also increases cancer cell growth (201). PER2 is also deregulated in cancer, its expression is lower in breast cancer tissue (201) and the knockout of *mPer2* gene increases cancer risk as it regulates DNA damage-responsive pathways (202). Wang et al. (203) suggested that PER2 overexpression inhibits cell proliferation by inhibiting PKB activation and enhances chemotherapeutic sensitivity via regulating genes downstream of the PI3K-PKB pathway. Additionally, Tokunaga et al. (204) discovered that a PER2 mutation leads to the downregulation of p53. On the other hand, there is evidence in both directions on a role for BMAL1 in cancer progression, probably indicating the heterogeneity in cancer itself. BMAL1 overexpression downregulates cyclin B, a cell cycle regulator, which leads to cancer cell proliferation (205). Similarly, in lung cancer, c-myc, a transcription factor present in tumor cells that regulates cell differentiation, has enhanced transcriptional activity with a loss of BMAL1 and PER2 (206). Jiang et al. (206) also showed the tumor-suppressing nature of BMAL1 as it binds to the p53 promoter region in pancreatic cancer. However, Elshazely et al. (207) discovered that BMAL1 is overexpressed in malignant pleural mesothelioma (MPM). In summary, these data demonstrate that the circadian clock plays a role in tumourigenesis, and given the overlap in pathways regulated by the SIK family and the clock, SIK could well be an interesting target that acts on both systems, providing a multipronged therapeutic approach.

### 2.6. The Role of SIK in Regulating Immune Function

Inflammation is a complex and coordinated response mounted by the host in response to tissue injury or infection, which aims to quickly isolate and remove the dangerous causative agent, repair any local damage, and restore tissue homeostasis. The acute inflammatory response is established minutes to hours after local sensing of infectious or inflammatory stimuli and is mediated by the innate immune system; a rapid and nonspecific first line of defense against infection, comprised of multiple physical barriers (skin and mucous membranes), chemical mediators (both pro- and anti-inflammatory), and immune cell populations (including monocytes, macrophages, neutrophils, dendritic cells, mast cells, and granulocytes) (208).

#### 2.6.1. Innate immune function.

A growing body of literature now demonstrates that the SIKs play a key role in innate immunity by regulating innate immune cell production of both pro- and anti-inflammatory cytokines; small, secreted proteins, including tumor necrosis factor-α (TNF-α) and the interleukins (ILs), that are central coordinators of the inflammatory response. However, there is conflicting evidence as to which SIK isoforms are involved in this process and whether their endogenous activity acts to enhance or inhibit proinflammatory signaling.

Some of the earliest reports examining the link between the SIKs and innate immune function found that these kinases act to suppress macrophage proinflammatory signaling. Macrophages sense pathogens via the recognition of conserved microbial motifs by specialized pattern recognition receptors (PRRs) (209). For example, the ligation of Toll-like receptor 4 (TLR4) by the bacterial cell wall component lipopolysaccharide (LPS) activates the transcription factor NF-κB to drive the production of proinflammatory mediators (210). Using the Raw 264.7 macrophage cell line, Yong Kim et al. (211) showed that the overexpression (OE) of SIK1 and SIK3, but not SIK2, resulted in decreased NF-κB activation after LPS simulation and that LPS-induced expression of the proinflammatory cytokines TNF-α and IL-6 was attenuated following OE of SIK1 or SIK3. Similar results were obtained by Sanosaka et al. (212), who also found that the OE of SIK3 in Raw 264.7 macrophages resulted in decreased expression of IL-6, IL-12, and inducible nitric oxide synthase (iNOS), following treatment with LPS (notably, however, the levels of TNF-α were unchanged). Conversely, the shRNA-mediated knockdown (KD) of both SIK1 and SIK3 in Raw 264.7 macrophages enhances LPS-driven NF-κB activation, and both bone marrow-derived macrophages (BMDMs) and thioglycolate-elicited peritoneal macrophages (TEPMs) generated from SIK3 knockout (KO) mice display increased IL-6, IL-12, and iNOS expression following LPS stimulation (again TNF-α remained largely unaffected) (212).

Interestingly, however, TEPMs from either SIK1 KO or SIK2 KO mice displayed no increase in proinflammatory mediator production when stimulated with LPS; in fact, their levels were slightly reduced (212). This discrepancy in the role of SIK1 in regulating LPS responses currently remains unsolved; however, differences in macrophage type and method of SIK ablation are likely to play a role. Indeed, the KD of SIK1 in microglia (the macrophages of the brain) exacerbates their expression of TNF-α, IL-1β, and IL-6 and enhances NF-κB signaling, following alcohol-induced inflammation, highlighting that SIK1 acts to limit the inflammatory response in this innate immune cell type (213). Significantly, this dampening of microglial inflammation via SIK1 regulation of NF-κB aligns with the mechanistic explanation provided by Yong Kim et al. (211) who propose that inhibition of TLR4 signaling by the SIKs is, in part, caused by the physical interaction of SIK1/3 with TAB2, which blocks TRAF6 ubiquitination (required for NF-κB signaling). However, despite finding increased proinflammatory signaling, Sanosaka et al. (212) did not find any differences in the activation of NF-κB pathway components downstream of TRAF6 in SIK3 KO macrophages. Therefore, future work is needed to uncover the underlying mechanism(s) and to fully understand which SIK isoforms, in which innate immune cells, may act to suppress proinflammatory signaling.

In stark contrast to the studies outlined above, a large body of literature exists demonstrating that the endogenous activity of the SIKs suppresses macrophage anti-inflammatory signaling. This was first demonstrated by Clark et al. (20) who found that the pharmacological blockade of the SIKs using the potent pan-SIK inhibitor HG-9-91-01 increased the production of the anti-inflammatory cytokine IL-10 in LPS-stimulated BMDMs. In addition, Ozanne et al. (214) found that the tyrosine kinase inhibitors bosutinib and dasatinib were potent SIK inhibitors, and both increased IL-10 production in LPS-treated BMDMs to the same level as HG-9-91-01. Notably, both studies found that treatment of LPS-stimulated BMDMs with SIK inhibitors caused their polarization toward an anti-inflammatory, regulatory phenotype (known as M2b), likely via autocrine IL-10 signaling (20, 214). Polarization toward the IL-10-secreting M2b phenotype also occurs when macrophages are costimulated with LPS and ligands that increase intracellular cyclic AMP; a classic example is prostaglandin E_2_ (PGE_2_) (215). Interestingly, MacKenzie et al. (216) found that the treatment of BMDMs with both LPS and PGE_2_ increased their production of IL-10, mirroring the effect seen with two different SIK inhibitors. The KD of CRTC3, but not CRTC1 or CRTC2, blocked the ability of PGE_2_ or SIK inhibitors to increase LPS-induced IL-10 expression in macrophages (20, 216), while the overexpression of a SIK2[S343A] mutant, which cannot be inactivated by PKA, or a SIK2[T96Q] “drug-resistant” mutant restricts CRTC3 to the cytoplasm and blocks SIK inhibitor-mediated IL-10 production, respectively (20, 214, 216), therefore suggesting that at least SIK2 negatively regulates macrophage IL-10 production via CRTC3. However, what about other SIK isoforms? In the ANA-1 macrophage cell line, the enhancement of LPS-driven IL-10 production and CRTC3 nuclear localization by HG-9-91-01 was blocked by the OE of SIK1, 2, or 3, suggesting that all three SIK members contribute toward IL-10 regulation (217). However, utilizing transgenic mice wherein each SIK is replaced with a kinase-inactive (KI) version, Darling et al. (85) demonstrated that it is SIK2 and SIK3, but not SIK1, that are the critical regulators of macrophage IL-10 production in response to LPS. In both fetal liver-derived macrophages (FLDMs) and BMDMs, the loss of SIK1 activity had little effect on LPS-driven IL-10 production, whereas SIK2 KI, SIK3 KI, or double SIK2/3 KI macrophages produced significantly higher levels of IL-10 after LPS stimulation in comparison to WT cells (85). Together these studies establish a model whereby the SIKs suppress anti-inflammatory macrophage signaling via CRTC3 phosphorylation to negatively regulate IL-10 production and anti-inflammatory M2b polarization (FIGURE 6).

In addition, the SIKs have also been found to regulate proinflammatory macrophage mediator production. HG-9-91-01 reduces the production of TNF-α and IL-6 in LPS-stimulated BMDMs (20), and escitalopram (traditionally a selective serotonin reuptake inhibitor) has been shown to inhibit SIK2 activity and block the LPS-driven production of TNF-α, IL-6, and IL-1β in Raw 264.7 macrophages (218). Furthermore, production of TNF-α, IL-6, and IL-12 was significantly lower in LPS-stimulated macrophages isolated from KI mice of all SIK isoforms (85), whereas the overexpression SIK1, 2, or 3 in ANA-1 macrophages reversed the inhibitory effect of HG-9-91-01 on LPS-induced expression of TNF-α and IL-12 (217). This strongly suggests that all three SIK isoforms act to regulate the production of proinflammatory cytokines. However, the mechanism by which this occurs is currently unclear, as it has been reported that SIK controls the phosphorylation of HDAC4 to regulate NF-κB signaling (218), whereas CRTC3 has been suggested to mediate the proinflammatory impact of SIK OE (217). Therefore, future work is needed to fully understand the mechanism(s) by which the SIKs control proinflammatory mediator production.

Macrophage biology and indeed multiple aspects of immune function are tightly governed by the circadian clock and sleep; this has been reviewed extensively (219, 220). Inflammatory cytokines including IL-6, IL-12, CCL5, and CXCL1 are robustly rhythmic in expression, and the level of their secretion in response to LPS is dependent on the clock (221). Importantly, these rhythms are abolished in clock gene knockout animals (221). The production of these cytokines is regulated by the activity of all three SIK isoforms, albeit in a manner that is yet to be fully resolved. SIK activity also regulates circadian rhythms and is also likely to be part of the system by which time-of-day is conveyed to macrophages, which is thought to be via the sympathetic nervous system (222), and thus catecholamine-GPCR signaling, which converges on CREB. Therefore, perhaps some of the discrepancies may be resolved when considering time-of-day effects and the interconnected signaling loops between SIK, the circadian clock, and macrophage function.

In addition to murine macrophages, the SIKs have been found to regulate inflammatory signaling in a range of other innate immune cells. Indeed, various SIK inhibitors have been shown to block LPS, zymosan (TLR2 ligand), or Pam_3_CSK_4_ (TLR1/2 ligand)-induced anti-inflammatory IL-10, and proinflammatory TNF-α, IL-6, and IL-12, production in the THP-1 human monocyte cell line (20), human primary monocytes and macrophages (20, 223, 224), bone marrow-derived dendritic cells (BMDCs) (20, 224), murine gut immunoregulatory myeloid cells (224), and human monocyte-derived DCs (223, 224). Furthermore, the SIKs have recently been demonstrated to regulate mast cell cytokine production. After stimulation with the potent mast cell activator IL-33, SIK pharmacological blockade inhibited the production of a range of cytokines [granulocyte-macrophage colony-stimulating factor (GM-CSF), IL-13, and TNF-α] and chemokines (the chemoattractant cytokines CCL2, 3, 4, and 24) (225). Interestingly, IL-33-dependent cytokine and chemokine secretion was unaffected in mast cells generated from SIK1 KI mice, slightly suppressed in SIK3 KI or KO mast cells, and fully blocked in SIK2/SIK3 double KI or KO mutants (225), demonstrating that SIK2 and 3 are key regulators of mast cell inflammatory mediator production.

Currently, it remains an open question as to why some studies demonstrate that the endogenous activity of the SIKs acts to inhibit innate immune cell proinflammatory signaling, while others strongly suggest that they instead act to promote pro- and inhibit anti-inflammatory signaling. However, almost all studies use different cell types, experimental conditions, and genetic manipulations of the SIKs (with all isoforms often not studied), therefore not allowing direct comparisons and an assessment of the role these factors play. Furthermore, SIK3 KO macrophages also secrete increased levels of IL-10 alongside proinflammatory mediators in response to LPS (212), and SIK2/3 KO mast cells produce increased levels of the chemokine CXCL2 following IL-33 stimulation (225), suggesting that a pro- or anti-inflammatory role for the SIKs need not be mutually exclusive. Therefore, future work replicating existing data, comparing innate immune cell types, and examining each SIK isoform is needed to fully understand the endogenous role of the SIKs in regulating innate immune cell function.

#### 2.6.2. Infection and inflammation in vivo.

**FIGURE 9.**
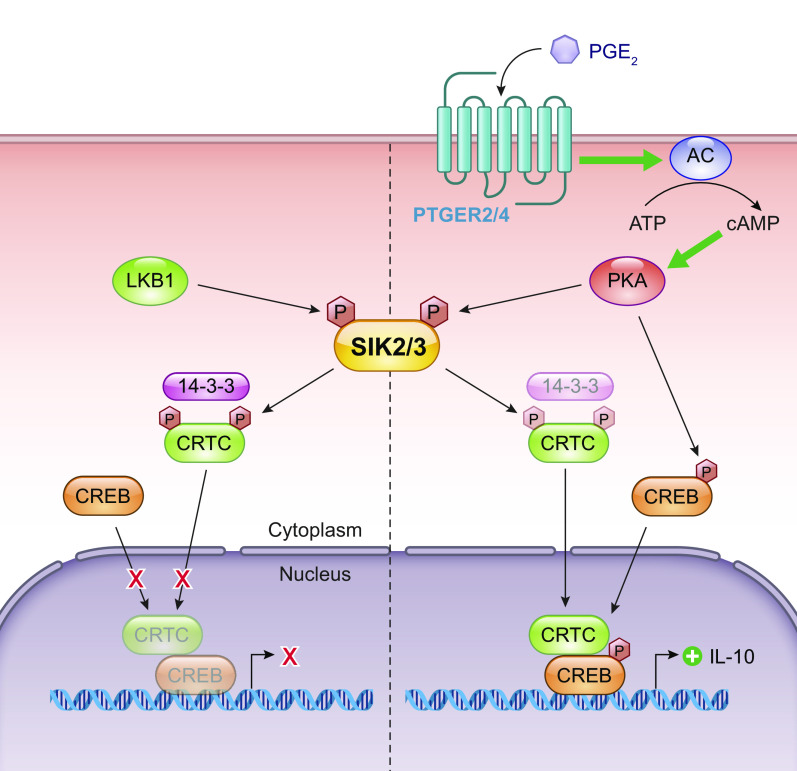
SIK-mediated regulation of inflammation. Under basal conditions, SIK2/3 are activated by LKB1 to sequester CRTC to the cytoplasm, maintaining a proinflammatory environment. Upon extracellular signals such as prostaglandin E_2_ binding to its receptor, PKA-mediated SIK phosphorylation allows the dephosphorylated CRTC to coactivate CREB, resulting in the transcription of anti-inflammatory genes such as IL-10. SIK, salt-inducible kinase; CRTC, cAMP response element binding protein (CREB)-regulated transcription coactivators; LKB1, liver kinase B1; Adapted from Ref. 5 with permission from *Trends in Endocrinology and Metabolism*.

In addition to their role in regulating cellular immune responses, the SIKs have also been demonstrated to regulate infection and inflammation in vivo. Recently, escitalopram (found to be a SIK2 inhibitor) was shown to have a beneficial effect in an LPS-induced acute lung injury model, reducing alveolar damage and edema, TNF-α and IL-6 production, and inflammatory immune cell infiltration (218). In addition, ARN-3236 (a SIK2-selective inhibitor) and dasatinib (shown to be a pan-SIK inhibitor) both decrease lung inflammation and fibrosis in a bleomycin-induced pulmonary fibrosis model, suggesting that multiple SIK isoforms may regulate inflammation in vivo (226, 227). Indeed, a recent study by Fu et al. (217) demonstrated that all three SIK family members regulate colonic inflammation. In both a 2,4,6-trinitrobenzene sulfonic acid (TNBS) and a dextran sulfate sodium (DSS)-induced colitis model, treatment with HG-9-91-01 attenuated weight loss, colon shortening, histological markers of disease severity, and myeloperoxidase activity (a marker of neutrophil infiltration). Furthermore, HG-9-91-01 treatment decreased colonic TNF-α and IL-12 levels and increased colonic IL-10 production in both colitis models. Strikingly, the OE of SIK1, SIK2, or SIK3 in the colon reversed the effect of HG-9-91-01 on TNBS-induced weight change, colitis severity, and TNF-α, IL-12, and IL-10 levels, demonstrating that the anti-inflammatory effect of HG-9-91-01 during colonic inflammation is dependent on all three SIK isoforms (217). Isolation of gut immune cells from TNBS- or DSS-treated mice found that HG-9-91-01 increased IL-10 production in colonic macrophages (but not neutrophils, DCs, T cells, or B cells), and the specific deletion of IL-10 in colonic macrophages blocked the therapeutic effect of HG-9-91-01 in both colitis models (217). Therefore, the SIKs control the in vivo production of IL-10 in colonic macrophages (corroborating the above in vitro macrophage studies) to regulate gut inflammation. Interestingly this is slightly at odds with the findings of Darling et al. (85) who found that only SIK2 and SIK3 regulate macrophage IL-10 production; however, differences in the inflammatory agent (TNBS/DSS vs. LPS), macrophage type (primary colonic macrophages vs. BMDMs/FLDMs), and genetic manipulation used (SIK OE vs. SIK KI mice) may account for this discrepancy. Nevertheless, these studies demonstrate that the SIKs act to promote inflammation in vivo (FIGURE 9) and highlight that their pharmacological blockade may be beneficial for treating multiple inflammatory diseases.

Conflictingly (and mirroring the cellular literature), some studies show that the SIKs act to suppress infection and inflammation, suggesting that their inhibition may actually be detrimental in certain situations. Indeed, SIK2 has recently been found to limit *Salmonella* infection; SIK2 KD in epithelial cells causes host cytoskeletal dysfunction, compromising *Salmonella*-containing vacuole integrity and resulting in cytosolic escape and bacterial hyperproliferation (50). Furthermore, the SIKs may limit airway inflammation via their action on Pannexin 1 (Panx1), an ATP-permeable channel. Both SIK1 and 2 were found to interact with Panx1, with SIK1 being shown to phosphorylate this channel at S205 causing its activation (228). HG-9-91-01 administration blocked Panx1 activation in vitro, and Panx1 S205A mutant mice displayed increased lung inflammation in a house-dust mite model of allergic airway inflammation, mirroring the phenotype of Panx1 KO animals (228). Finally, SIK3 has been demonstrated to limit endotoxemia in vivo, as intraperitoneal injection of LPS into SIK3 KO (but not SIK1 or SIK2 KO mice) animals resulted in increased mortality and serum levels of TNF-α and IL-6 (212). Notably, this higher mortality and serum IL-6 was also seen when animals lacking SIK3 only in the hematopoietic compartment were challenged with LPS, strongly suggesting that SIK3 activity protects against endotoxic shock by dampening proinflammatory signaling in myeloid cells. This contrasts with the above finding that SIK2 inhibition attenuates LPS-induced lung inflammation in vivo, suggesting that the role each SIK plays during inflammation is highly context dependent. Together, these studies show that the SIKs can also act to limit inflammatory signaling in vivo and highlight how future work is needed to fully untangle when, where, which, and how each SIK isoform regulates the inflammatory response.

Sleep and circadian rhythm disruption (SCRD) is a well-established risk factor for respiratory viral infections (229, 230) and also severe COVID-19 (231–233). This is unsurprising as the immune system is under tight sleep and circadian control. Leukocyte trafficking, host-pathogen interaction, and immune cell activation display diurnal rhythms (220), and circadian differences in response to pathogens are well documented (219). The infectivity of multiple viruses, including influenza, is dependent on the time of day (230, 234). Importantly, knockout of clock genes in mouse models increases proinflammatory cytokine levels, perturbs immune cell function and trafficking (219), and promotes the replication of a wide range of viruses (234, 235). Sleep disruption also leads to immune dysfunction, reducing natural killer cell activity (236), modifying proinflammatory cytokine production (70, 237, 238), and altering immune-related gene expression in multiple tissues (239), including the mouse brain (240), liver (241), and lung (69, 242). A similar disruption of the circadian clock and immune system is seen in blood samples from sleep-deprived human subjects (243, 244). The molecular determinants of how circadian disruption causes increased infectivity remains unknown. However, an analysis of all these transcriptomes from sleep-deprived subjects shows the upregulation of *Sik1* in common, and SIK1 has also been identified as a critical host factor that determines SARS-CoV-2 infectivity as identified from a genome-wide CRISPR loss-of-function screen (245). This remains a correlation, but the accumulating evidence suggests overlapping pathways. A study of viral infectivity and its circadian control in the SIK loss-of-function mutants would indeed be very interesting.

#### 2.6.3. Adaptive immune function.

Several recent reports have found that the SIKs act to regulate adaptive immunity. The adaptive immune system, comprised of T and B lymphocytes, allows the generation of a highly pathogen-specific, cell-mediated (T cells), and antibody-mediated (B cells) immune response, which neutralizes the invading pathogen and generates immunological memory allowing for a rapid and augmented response upon reinfection (246). However, contrasting with myeloid cells, the SIKs do not seem to regulate cytokine production in lymphocytes, as treatment with HG-9-91-01 has no effect on IL-10 production in both T and B cells (217, 224) but instead acts to control lymphoid linage determination, differentiation, and survival. The ectopic overexpression of MEF2C (whose activity is known to be SIK controlled) in early thymocyte progenitor acute lymphoblastic leukemia promotes B-cell development and inhibits early T-cell differentiation (247). Treatment of the LOUCY acute lymphoblastic leukemia cell line with HG-9-91-01 restored normal T-cell development (247), therefore suggesting that the SIKs impact lymphoid lineage decision-making via regulating MEF2C activity. Indeed, utilizing a combination of SIK KO and SIK KI mice, Nefla et al. (248) demonstrated that SIK2 and SIK3 are critical regulators of in vivo T-cell development. While SIK2 KI or SIK1/2 double KI mutants had normal T- and B-cell compartments, SIK3 floxed/Vav-iCre animals (where SIK3 is genetically deleted only in hematopoietic cells) displayed slightly reduced T-cell numbers in the spleen and lymph nodes, while B-cell numbers remained unaffected (248). Furthermore, SIK2 KO/SIK3 floxed/Vav-iCre double mutant animals had normal peripheral B-cell numbers but drastically reduced T-cell counts in the thymus, spleen, and lymph nodes, with most of any remaining cells displaying an immature phenotype (248). Interestingly, the SIK2/3 mutant animals also had decreased regulatory T-cell (Tregs, an immunoregulatory T-cell subset) numbers in peripheral lymphoid tissues, mirroring the finding that LKB1 KO mice have severely depleted Tregs and that coincubation of Tregs with both SIK and MAP/microtubule affinity-regulating kinase inhibitors decreases Treg survival (249). Together, these data demonstrate that the SIKs impact the adaptive immune system by regulating T-cell linage commitment, differentiation, and survival.

### 2.7. Bone Development

The role of SIK in bone development centers on its regulation of parathyroid hormone (PTH) and parathyroid hormone-related peptide (PTHrP) signaling, peptide hormones that maintain blood calcium levels (250). PTH is released in response to hypocalcemia and stimulates the release of calcium from the bones, where the vast majority of calcium in the body is stored. PTH subsequently stimulates calcium resorption from bone and bone formation. PTH receptors (PTH1 receptor) are most highly expressed in osteocytes, which are also the most abundant cell type in the bone (251). Activation of these receptors, which are G-protein coupled, leads to PKA signaling and the CREB-dependent expression of receptor activator of NF-κB ligand (RANKL) (252) and the MEF2-mediated suppression of sclerostin transcription (39, 253); sclerostin is an inhibitor of bone formation. On the other hand, RANKL is a cytokine and the primary mediator of PTH-stimulated bone resorption (254). Thus PTH1 receptors have become a focus for treatments for osteoporosis (250). PTH signaling also leads to SIK2 phosphorylation by PKA, thus reducing the phosphorylation of HDAC4/5 and CRTC2 (269) and inhibiting MEF2-mediated and potentiating CREB-mediated transcription. Therefore, SIK2 inhibition would be predicted to have PTH-like effects and promote bone formation. In agreement, the pan-SIK inhibitor YKL05099 led to reduced HDAC4/5 phosphorylation and CRTC2 nuclear translocation. This coincided with PTH-like gene expression changes, including upregulation of RANKL and suppression of sclerostin expression in osteocyte cultures. Once daily treatment with the small molecule SIK inhibitor YKL-05-099 increases bone formation and bone mass but, surprisingly, also led to a reduction in osteoclast numbers and activity and reduced bone resorption (255), suggesting other effects of either YKL-05-099 or pan-SIK inhibition that could not be resolved in this study.

Gene knockout models have added some detail to this picture to suggest the different SIKs do not perform equivalent functions, with each of the SIKs regulating the specific aspects of physiology in the different bone cell types. Inducible, global deletion of SIK2 or SIK3 in mice results in increased bone mass, increased bone formation, and, distinct from the effects of YKL-05-099, increased bone resorption in hypogonadal female mice. YKL-05-099 also inhibits CSF1R, the receptor for the osteoclastogenic cytokine M-CSF in myeloid cells, and this could explain the antiresorptive effects of this drug (256). Since bone formation and resorption are typically coupled, current anabolic osteoporosis therapies also cause bone destruction. YKL-05-099 and thus dual targeting of SIK2/3 and CSF1R offer the opportunity to induce bone formation uncoupled from bone resorption (256).

Multiple studies have reported that SIK3 knockout or inactivation (*Sik3* T163A) results in small size and defects in skeletal development (63, 85). In the SIK3 KO, it was shown that this was due to severely delayed chondrocyte hypertrophy, which is essential for ossification. SIK3 is required for the phosphorylation and sequestration of HDAC4, thereby releasing MEF2C to facilitate hypertrophy through directed transcriptional pathways. Accordingly, SIK3 KO mice show accumulation of chondrocytes in multiple bones, an expansion of the growth plate and cartilage, and impaired skull bone formation, and many of these phenotypes were rescued by targeted expression of *Sik3* in chondrocytes. PTHrP acts on chondrocytes to block their development and hypertrophy, thus PTHrP-null mice do not survive postbirth, due to respiratory failure caused by a small circumference of the rib cage in association with extensive chondrocyte hypertrophy (257). However, the ablation of *Sik3* in chondrocytes rescues perinatal lethality in these mice (258) or indeed by *Hdac4/5* KO in these cells (259). The *Sik1* and *Sik2* single and double chondrocyte-specific KOs (cKOs) all exhibited normal growth plate phenotypes, indicating that Sik3 is the predominant isoform regulating chondrocyte differentiation, but *Sik1*/3 and *Sik2*/3 double cKOs show further delays in chondrocyte hypertrophy (258, 259), suggesting SIK1 and SIK2 can compensate for SIK3 to some extent. In this study, all three *Sik* single knockouts showed a normal skeletal phenotype; however, *Sik2*/3 double knockouts in osteoblasts and osteocytes cause a high bone mass and accelerated bone turnover, which were rescued by the simultaneous deletion of HDAC4/5. SIK1 has also been shown to inhibit the proliferation and differentiation of osteoblast precursors, within SIK1 KO mice displaying higher bone mass, osteoblast number, and bone formation rate (260).

The increase in chondrocyte number and cartilage growth associated with SIK3 KO/inhibition (63) could be beneficial for the treatment of osteoarthritis, which is associated with reduced cartilage thickness. To overcome the deficits associated with global SIK3 KO, Yahara et al. (261) used tamoxifen-induced conditional chondrocyte-specific *Sik3* knockout mice to show that these mice are resistant to osteoarthritis. They also showed that pterosin B, identified from a high-throughput screen as a SIK3 inhibitor, leads to reduced MEF2C activity and increased CRTC2 activity and protects mice from the development of osteoarthritis following destabilization of the medial meniscus (DMM) surgery (261).

**FIGURE 10.**
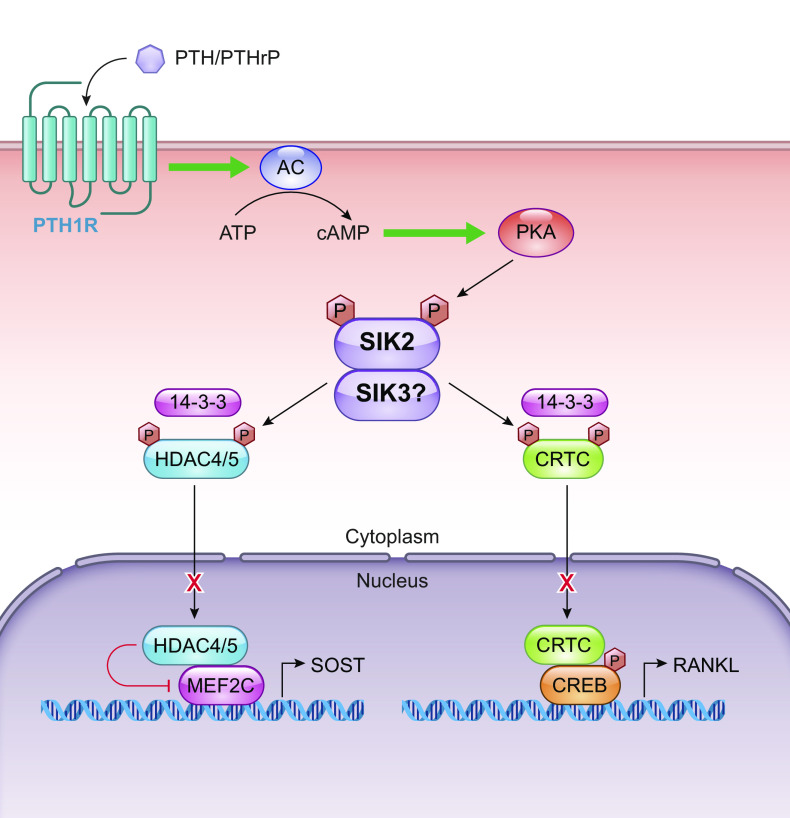
The role of SIK2 in regulating bone formation. Activated SIK2 (and also SIK3) inhibits CREB-mediated transcription and relieves HDAC-mediated MEF2C repression, leading to increased bone resorption. SIK, salt-inducible kinase; LKB1, liver kinase B1; CRTC, cAMP response element binding protein (CREB)-regulated transcription coactivators; HDAC, histone deacetylase; PTH, parathyroid hormone; PTHrP, parathyroid hormone-related peptide. Adapted from Ref. 5 with permission from *Trends in Endocrinology and Metabolism*.

In summary, SIK1-3 regulates diverse aspects of bone and cartilage formation and function (FIGURE 10), and thus SIK inhibitors provide exciting new avenues for therapies in osteoporosis and osteoarthritis.

### 2.8. Skin Pigmentation

Melanin is the pigment responsible for hair and skin color and is produced within specialized cells in the skin known as melanocytes and subsequently distributed to surrounding epidermal keratinocytes (262). Melanin shields the underlying cells from excessive ultraviolet (UV) penetration and is thus an important defense against UV-induced DNA damage, which is the leading cause of skin cancers including melanoma (263). UV exposure triggers the production of melanin, which itself occurs in two forms; the darker eumelanin, which is the main determinant of pigmentation, and the reddish yellow pheomelanin, which provides relatively less protection against UV damage and reactive oxygen species. The melanocortin 1 receptor (Mc1R), one of the first genes identified to regulate skin color, is a G_s_ protein-coupled receptor that regulates melanin production, and its activation directs the switch from pheomelanin to eumelanin (262). Mc1R is highly polymorphic in northern European populations, with loss-of-function variants associated with red hair and fair skin (264), poor tanning ability, and an increased risk for melanoma (263), but *Mc1R* shows almost no functionally significant variation among African populations (262). Exposure to UV light results in DNA damage in keratinocytes, which in turn triggers p-53-mediated transcription of the pro-opiomelanocortin gene (POMC gene), and POMC peptides are proteolytically cleaved to produce an α-melanocyte-stimulating hormone (α-MSH) and adrenocorticotropic hormone (ACTH), both of which are ligands at the Mc1R receptor (265, 266). α-MSH is secreted to stimulate the Mc1R receptor on melanocytes, which activates adenylate cyclase. The resulting elevation in cAMP triggers PKA-mediated phosphorylation of CREB at Ser-133 to ultimately increase the CREB-mediated transcription of microphthalmia-associated transcription factor (MITF) (267). MITF activates the transcription of *Tyrosinase* and related enzymes, which are the rate-limiting steps in the synthesis of eumelanin from tyrosine (262). MITF also increases the production of other melanosomal components and trafficking machinery, ultimately resulting in increased melanin production and export (268). Pharmacological activation of this pathway with forskolin and synthetic α-MSH analogs to achieve sunless tanning has been explored (268). Given the well-established role of CRTC/SIK signaling in the regulation of CREB transcription downstream of ACTH, a role for this pathway, and thus SIK inhibitors, in regulating melanogenesis has also been considered. Horike et al. (269) showed that all three SIKs and CRTCs are expressed in melanoma cells and that all CRTCs are capable of stimulating CREB-mediated transcription of MITF, but their subsequent work focussed on CRTC1 and SIK2. UV light caused the nuclear localization of CRTC1 and the overexpression of SIK2 inhibited MITF transcription. Agouti mice *A^y^/a* have yellow hair due to the overproduction of agouti protein, which is an antagonist at Mc1R and results in increased pheomelanin production (270), and the application of forskolin topically in these mice rescues the phenotype and results in brown pigmentation in hair follicles (271). The complete knockout of SIK2 on this genetic background changes coat color to brown, indicating SIK2 is a major determinant of pigmentation in mice (269). However, wild-type back coloration was not achieved, indicating other SIKs may also play a role. In agreement, HG-9-91-01, which targets all three SIK isoforms when applied topically to the skin of red-haired mice, which carry an inactivating mutation in Mc1R (Mc1re/e), increases transcription of MITF and the transfer of melanosomes to keratinocytes, thus significantly darkening skin (272). Interestingly, this did not require exposure to UV light, but the upregulation of MITF in response to SIK inhibitors in vitro was dependent on the activity of LKB1. The more skin-permeant second-generation inhibitors YKL-06-061 or YKL-06-062 also showed a similar effect (272). The effect of any of these compounds in protecting from UV-induced carcinogenesis is yet to be demonstrated.

## 3. PHARMACOLOGICAL TARGETING OF SIKS

**FIGURE 11.**
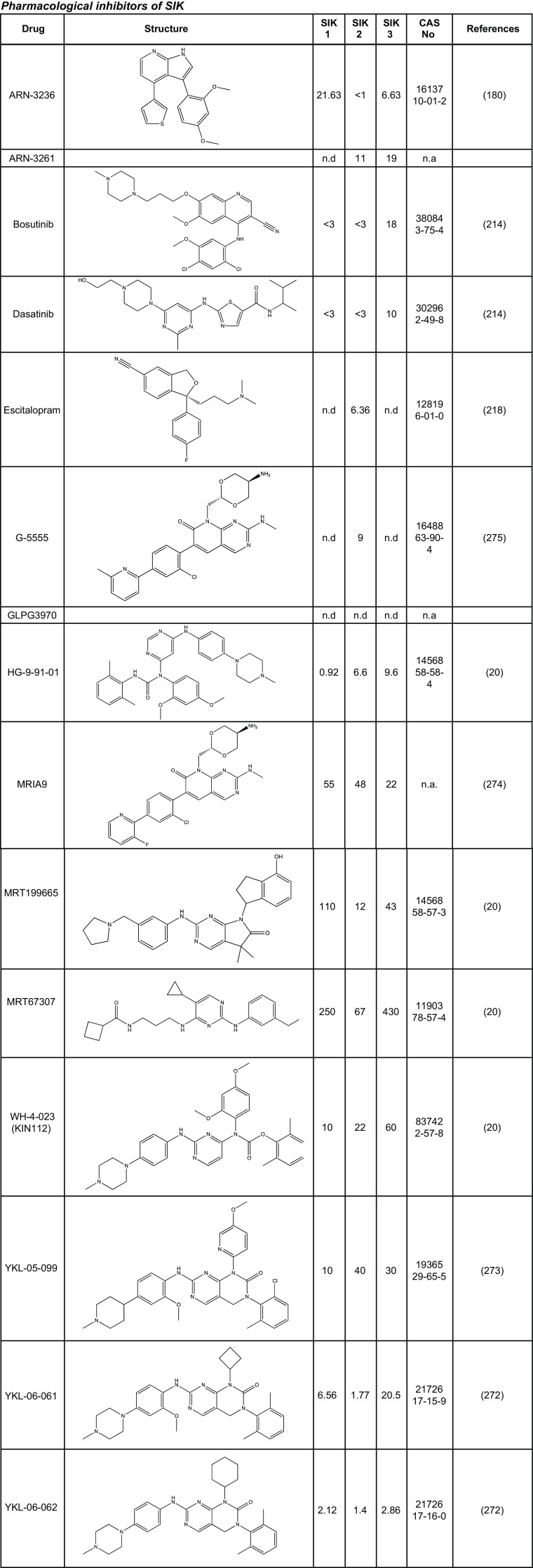
Pharmacological inhibitors of salt-inducible kinase (SIK) (see Refs. 20, 214, 218, 223, 272–275).

To understand the role of SIKs in various aspects of physiology, SIK inhibitors have been developed (FIGURE 11) to help investigate the biological functions of this family away from genetic manipulation and gene redundancies. One widely used pan-SIK inhibitor is HG-9-91-01 (which inhibits SIK1, SIK2, and SIK3 at an IC_50_ of 0.92 nM, 6.6 nM, and 9.6 nM, respectively). HG-9-91-01 is predominantly serum bound and has a very short half-life due to its rapid degradation by mouse liver microsomes (half-life of 11 min), which makes its use in vivo limited (273). Nevertheless, HG-9-91-01 has been very important in elucidating the physiological roles of SIK in several studies. These include the use of small molecule SIK inhibitors to produce an anti-inflammatory phenotype in macrophages. SIK inhibitors significantly increase the production of the anti-inflammatory cytokine interleukin (IL)-10 and reduce the secretion of proinflammatory cytokines like TNF-α (20, 85) in macrophages. HG-9-91-09 was used by two different studies to demonstrate the regulation of insulin secretion from pancreatic islets (135, 136); however, the two studies reported conflicting results. Sakamaki et al. (136) showed that HG-9-91-01 reduced glucose-stimulated insulin secretion in islets similarly to knockdown of *Sik2*. Kim et al. (135) showed an increase resulting from HG-9-91-01 application in islets, and that this was dependent on *Sik1*. HG-9-91-01 has also been used to demonstrate the role of SIK in sleep regulation. As HG-9-91-01 is not brain penetrant, Wang et al. (48) administered the compound via intracerebroventricular injection and demonstrated reduced NREM sleep and reduced phosphorylation of those synaptic proteins implicated in the regulation of sleep, but described these proteins as AMPK targets, which is surprising given this compound does not inhibit AMPK.

HG-9-91-09 targets the ATP-binding site in SIK. In addition, a threonine residue within the gatekeeper site permits access to many different pharmacophores and nucleotides to the active site. This small amino acid-derived hydrophobic pocket adjacent to the ATP-binding site is unique to the SIKs within the AMPK-related kinase family but is also present within multiple tyrosine kinases as explained below. Mutation of this site abolishes sensitivity to the inhibitor but retains SIK activity. This mutant was used to validate the role of SIK in gluconeogenic suppression in the liver (142). Therefore, HG-9-91-01 does not inhibit any non-SIK AMPK-related kinase family members but does inhibit protein tyrosine kinases including Src, Lck, and Yes, which also possess a threonine residue in the gatekeeper site. KIN112 or WH-4-23 is another compound structurally related to HG-9-91-01 and a potent inhibitor of SIK as well as Lck and Src tyrosine kinases, and compounds that target the tyrosine kinases dasatinib and bosutinib also potently target SIK (214). For this reason, Darling et al. (8) recommend the simultaneous use of a structurally unrelated inhibitor such as MRT199665 to avoid confounds due to off-target effects. MRT199665 is specific to SIKs and inhibits SIK1, SIK2, and SIK3 at IC_50_ values of 110 nM, 12 nM, and 43 nM, respectively, but also inhibits other AMPK-related kinases. MRT1996655 and its predecessor MRT67307 have been used in multiple studies to elucidate SIK function in immune regulation. MRT67307 was developed as an inhibitor of the IκB kinase (IKK)-related kinases TBK1 and IKKε, but its effects of increasing IL-10 production and suppressing proinflammatory cytokines in macrophages were found to be through the inhibition of SIK (20). MRT1996655 is structurally related and a potent inhibitor of SIKs, MARKs, and AMPK but not of TBK1 and IKKε (20) and has been used to demonstrate that inhibition of SIK potentiated apoptotic cell death induced by transforming growth factor-β stimulation (276), and also program an anti-inflammatory phenotype in macrophages (85). As such, SIK inhibitors have shown therapeutic potential for the treatment of inflammatory and autoimmune diseases (85) and several trials with SIK inhibitors are ongoing in this space. Galapagos NV has a series of SIK2/3 inhibitors of which GPLG3970 is the most clinically advanced and been shown to be safe and well tolerated but failed to improve outcomes over placebo in either rheumatoid arthritis or ulcerative colitis. Other compounds related to GPLG3970 are being developed.

A SIK inhibitor called YKL-05-099 was developed by Sundberg et al. (273) by using HG-9-91-09 as a starting point and has shown higher selectivity for SIKs and improved pharmacokinetic properties, achieving >16 hours of free serum concentrations above its IC_50_ and is well tolerated in vivo. These properties make it a useful tool widely used in animal experiments and to provide proof-of-concept in the treatment of disease. Wein et al. (255) have shown that in vivo inhibition of SIK using a single daily dose of YKL-05-099 can mimic the effects of the parathyroid hormone on osteocytes, increasing bone formation and bone mass, thus demonstrating the potential of small molecule SIK inhibitors in the treatment of osteoporosis. YKL-05-099 attenuated disease progression and extended animal survival in a mouse model of acute myeloid leukemia through inhibition of the SIK3-HDAC4-MEF2C axis (277). Two further second-generation SIK inhibitors also developed from HG-9-91-01 when applied topically to mouse skin and human breast tissue resulting in upregulated MITF production and melanogenesis (272). These compounds are being developed by the SME Soltego for application in the protection of skin from UV damage.

ARN-3261 and ARN-3236 are orally bioavailable highly specific SIK inhibitors developed by Arrien Pharma. ARN-3261 preferentially targets SIK2 and SIK3 (IC_50_ of 11 and 19 nM, respectively) and has been shown to sensitize ovarian cancer cell lines and xenografts to carboplatin (181) and is currently in Phase 1a/1b clinical trials in patients with ovarian, peritoneal and other solid tumors (278). MRIA9, a SIK inhibitor developed from the p21-activated kinase (PAK) inhibitor G-5555, was also shown to be effective on an ovarian cancer model and is in further development (274). ARN-3236 is also blood-brain barrier permeant (118) and represents a useful research tool for studies on SIK function in the brain. ARN-3236 demonstrates antidepressant-like properties in chronic stress mouse models, and its mechanism of action was proposed to be the inhibition of the SIK2-CRTC1-CREB-BDNF pathway in the hippocampus. However, given the low translatability of mouse models in psychiatric conditions and as multiple complex pathways play into the pathogenesis of depression, the exact mechanisms and the therapeutic relevance remain to be determined. It also remains to be seen whether ARN-3236 regulates circadian activity, where few small molecules that are brain permeant have been identified. SK-124 is a nanomolar SIK2/3 inhibitor that is orally bioavailable and was recently developed using a structure-based drug design followed by an iterative medicinal chemistry approach. When administered daily to mice, SK-124 increased blood calcium and vitamin D, in addition to increased bone formation and bone mass. Importantly, no short-term toxicity was evident (279).

Targeting the SIK family is evidently an exciting area with a wide scope for therapeutic potential; however, a possible liability for the development of SIK inhibitors lies in the pivotal role of these kinases in glucose and lipid metabolism, steroidogenesis, and microtubule function (see sect. 2.3), which may introduce unwanted side effects when administered in vivo. Therefore, it is of great importance to further examine the effects of newly developed SIK inhibitors in vivo to fully elucidate the physiological consequences their inhibition might have.

## 4. CONCLUSIONS AND FUTURE DIRECTIONS

In summary, the members of the SIK family are emerging as master regulators of multiple aspects of physiology, including metabolism, immune function, development, and sleep and circadian rhythms. As SIKs are unique in their ability to be exquisitely modulated by extracellular signals, the SIKs serve to alter transcriptional and posttranscriptional responses to inputs that modify physiology. Sleep and circadian rhythms are master regulators of physiology, and it is emerging that the SIK family is at the hub of the molecular regulation of these processes. Given the widespread actions of SIK in other domains of physiology, it is likely that there is mechanistic overlap at the level of SIK as to how sleep and circadian rhythms regulate other aspects of physiology and how these systems feed back into the clock. Much remains to be understood about how each member of the SIK family is uniquely regulated by different inputs, how their protein- and phosphosite-level targets are specific to each family member and common, where they are redundant in function, and where they are unique. Future research will address these important questions, thus providing the substrate with which this important family of kinases can be developed for therapeutic application.

## GRANTS

A. Jagannath is supported by Biotechnology and Biological Sciences Research Council Grant BB/N01992X/1. R. G. Foster is supported by Wellcome Trust Grant WT106174/Z/14/ZMA.

## DISCLOSURES

No conflicts of interest, financial or otherwise, are declared by the authors.

## AUTHOR CONTRIBUTIONS

L.T., Y.R., and S.V. prepared figures; A.J., L.T., Y.R., Z.W., K.A., and S.V. drafted manuscript; A.J., Y.R., and S.V. edited and revised manuscript; R.G.F. approved final version of manuscript.
